# Highly Efficient and Environmentally Stable Radiative Cooling Fabric: Integrating Photoluminescence and Hierarchical Core–Shell Fibers

**DOI:** 10.1002/advs.75252

**Published:** 2026-04-13

**Authors:** Hongtao Liu, Hui Li, Yining Wang, Wei Sun, Zhuan Chen, Yongping Hou, Yongmei Zheng, Xuejian Chen, Bin Yu

**Affiliations:** ^1^ Key Laboratory of Bioinspired Smart Interfacial Science and Technology of Ministry of Education School of Chemistry Beihang University (BUAA) Beijing P. R. China; ^2^ School of Transportation Science and Engineering Beihang University (BUAA) Beijing P. R. China; ^3^ Key Laboratory of Road and Traffic Engineering of the Ministry of Education College of Transportation Tongji University Shanghai P. R. China; ^4^ State Key Laboratory of Advanced Waterproof Materials Beijing Oriental Yuhong Waterproof Technology Co., Ltd Beijing P. R. China

**Keywords:** environmental stability, hierarchical core–shell architecture, photoluminescence, radiative cooling, UV‐resistant

## Abstract

Polymer‐based radiative coolers are promising for zero‐energy heat management due to their flexibility and processability. However, their cooling performance and outdoor durability must be enhanced, specifically in weatherability, mechanical robustness, and anti‐fouling properties. Here, we demonstrate a scalable radiative cooling polymer fabric based on a multi‐layer assembly structure and photoluminescent material integration. This fabric exhibits high radiative cooling efficiency and notable environmental stability, demonstrating its potential as a candidate material for applications in energy‐efficient building cooling and personal thermal management. The hierarchical core–shell architecture synergizes with photon‐manipulating photoluminescence to optimize solar radiation blocking and thermal re‐emission, achieving a remarkable effective solar reflectivity (ESR, 101.1%) in the specific spectral band. Combined with its exceptional average mid‐infrared emissivity (95.34%), our multi‐layer core–shell radiative cooling fabric (Mc‐sRCF) delivers a maximum daytime sub‐ambient cooling of 10.0 °C under Beijing summer conditions (peak solar intensity of 858 W·m^−^^2^) while providing a cooling power contribution of 83.78 W·m^−^^2^. Notably, the multi‐level fiber architecture, synergistically integrated with stable chemical bonds on the outer layer and embedded multi‐scale nanoparticles (TiO_2_), endows the fabric with superior mechanical robustness (tensile strength: 8.7 MPa), long‐term UV resistance, rainproof/anti‐fouling properties, and self‐extinguishing flame retardancy, offering a promising pathway for durable outdoor applications.

## Graphical abstract

A scalable radiative cooling fabric engineered with a multi‐layer assembly core–shell structure and integrated photoluminescent materials is reported. The fabric exhibits remarkable environmental stability, including UV resistance, mechanical durability, hydrophobicity, stain resistance, thermal stability, and flame retardancy, demonstrating its potential as a candidate material for applications in energy‐efficient building cooling and personal thermal management.

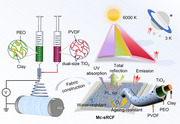

## 1 Introduction

Rational energy management is pivotal to reducing resource waste and carbon emissions. Under scientifically optimized energy control systems, human living and production environments have been significantly improved, with enhanced public health and rising well‐being indices. In the field of solar energy utilization, technologies such as visible‐light photovoltaics [1, 2], photothermal conversion [3, 4], and photoluminescence [5, 6] have achieved remarkable progress, while mid‐/far‐infrared stealth and heating applications are increasingly mature [7, 8]. Recently, radiative cooling technology, enabling zero‐energy‐consumption cooling through full‐spectrum solar management, has emerged as a global research hotspot [9, 10, 11], with consensus established on its fundamental principles [12], technical approaches [13], and material design [14, 15]. However, the critical bottleneck lies in achieving ultrahigh solar reflectivity while ensuring long‐term stability under harsh outdoor conditions [16, 17, 18]. Thus, developing radiative cooling materials with exceptional sunlight reflectivity, mechanical robustness, and weather resistance is the key to unlocking their real‐world applications.

Porous or nanoparticle‐embedded integrative radiative coolers are recognized as the most scalable material systems for practical deployment owing to their facile processing, low cost, and high solar reflectivity, exhibiting significant application potential [19]. Among these, electrospun nanofiber fabrics stand out by leveraging their light‐scattering fibrous networks and high‐density reflective pores to achieve exceptional solar reflectivity [20, 21, 22], while their unique fiber network structure effectively addresses the widespread issue of nanoparticle agglomeration in traditional composite materials [23, 24]. More importantly, through the precise selection and optimization of components, the fabricated specific fabric exhibits high absorption in the mid‐infrared band, enabling passive heat dissipation to the cold universe through the atmospheric transparency window for highly efficient radiative cooling [25]. Nevertheless, conventional fabrics composed of homogeneous material suffer from a low effective solar reflectivity (ESR) due to insufficient photon scattering at a single interface, and their unilateral characteristics fail to simultaneously meet the requirements of diverse environments [26]. Additionally, the environmental stability of polymeric radiative cooling materials has long been a critical challenge to be addressed in this field [27, 28]. For example, relevant studies have demonstrated that the incorporation of semiconductor materials (e.g., TiO_2_ particles) can significantly enhance the UV‐resistant performance of the materials [29, 30, 31]. However, such particles are difficult to disperse uniformly in the polymeric matrix, which inevitably leads to uneven stress distribution inside the materials, affecting the mechanical properties. Therefore, the development of high‐efficiency radiative composite fabrics with environmental stability becomes even more crucial.

The architecture of hierarchical core–shell fibrous structures, leveraging refractive index contrasts between constituent layers, profoundly amplifies solar reflectivity through multi‐interface light reflection mechanisms, thus boosting the cooling capabilities of radiative cooling fabrics. In addition, spectral redistribution allows for the precision engineering of the spectral response, optimizing solar reflectivity in desired regions. Photoluminescence (PL) is a non‐thermal‐equilibrium radiative process, i.e., materials absorb photons via electronic transitions and re‐emit lower‐energy photons after relaxation [32, 33, 34, 35]. This photon‐repurposing effect, which converts ultraviolet (UV) photons into visible wavelengths, decouples solar spectral management from thermal dissipation pathways, enhances solar reflectivity, and thereby improves the radiative cooling performance of the material. The hierarchical structural innovation and the associated photoluminescent behavior work synergistically to enhance solar reflectivity, possibly offering a critical pathway for improving the cooling performance of radiative cooling fabrics and providing key design principles for future radiative coolers.

Herein, we present a durable and highly efficient environmentally stable multi‐layer core–shell radiative cooling fabric (Mc‐sRCF) through hierarchical core–shell structural design and utilization of photoluminescent behavior via a straightforward one‐step coaxial electrospinning process. Multi‐interface reflection and photon redistribution via hierarchical core–shell structure and photoluminescence optimize solar spectrum management, resulting in a maximum ESR of 101.1%. Owing to the C ‐ F bonds in the outer Polyvinylidene fluoride (PVDF) layer and the C‐OH and C‐O‐C groups originating from the inner polyethylene oxide (PEO) layer, the fabric also exhibits a high average mid‐infrared emissivity of 95.34%. Furthermore, multi‐scale TiO_2_ particles are introduced into the spinning dope to resolve dispersion issues of nanoparticles, which leads to outstanding UV resistance and solar reflectance property through complementary absorption and reflection mechanisms. This hierarchical design, combined with intrinsic material properties, not only enhances radiative cooling performance but also improves environmental stability, including mechanical robustness, anti‐aging capability, and rainproof/anti‐fouling properties.

## 2 Result and Discussion

### 2.1 Design of Environmentally Stable Mc‐sRCF for High‐Efficiency Radiative Cooling

**FIGURE 1:**
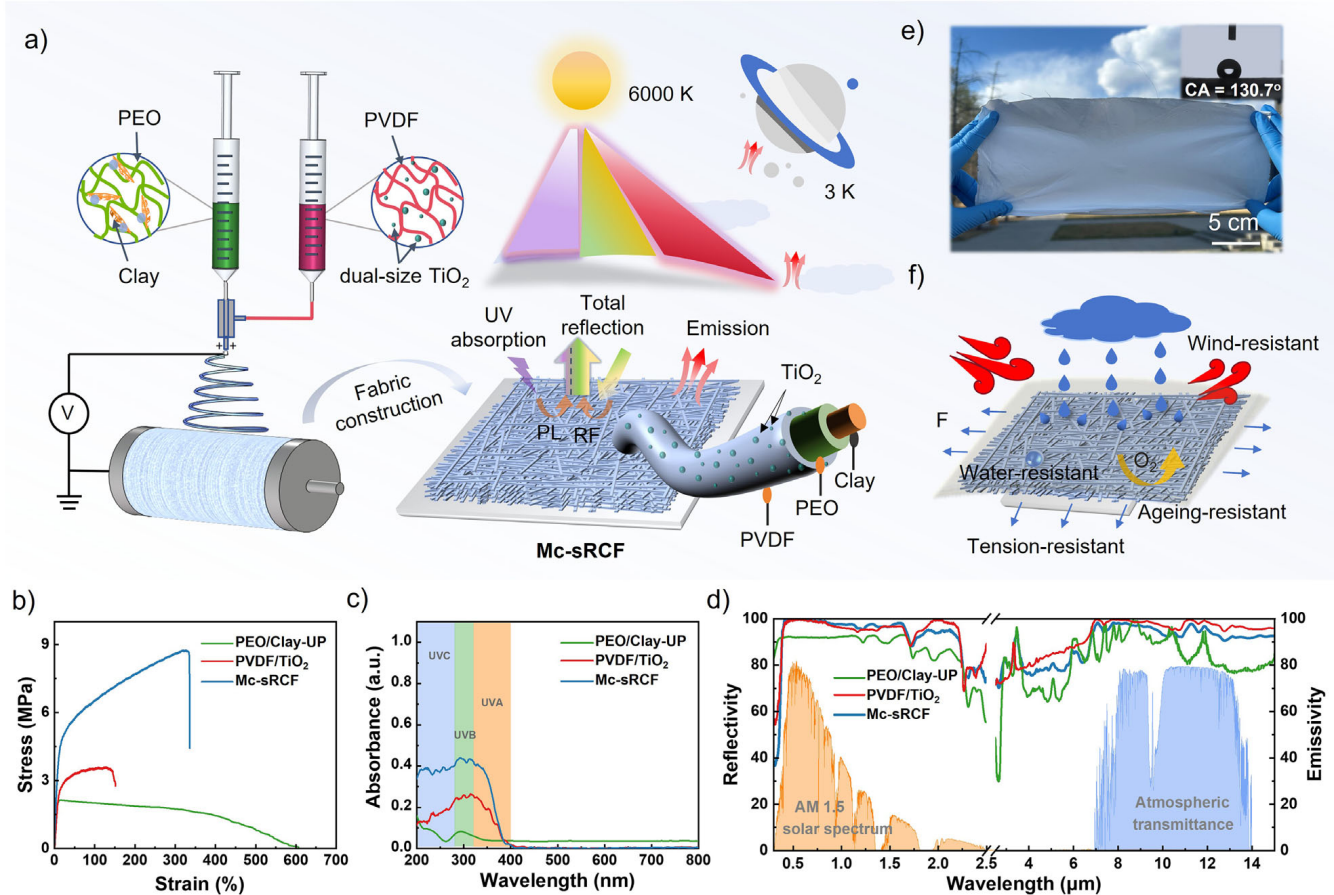
Design and key performances of the environmentally stable radiative cooling Mc‐sRCF. (a) Schematic illustration of the fabrication process and radiative cooling mechanism of Mc‐sRCF. (b) Stress‐strain curves comparing the mechanical properties of PEO/Clay‐UP fabric, PVDF/TiO_2_ fabric, and Mc‐sRCF. It reveals that Mc‐sRCF possesses the highest tensile strength along with an optimized fracture strain. (c) UV absorbance spectra of different fabrics. The result demonstrates that Mc‐sRCF possesses the superior UV‐absorption characteristic among these fabrics. (d) Full‐spectrum optical performance shows high solar reflectivity (particularly in the solar spectrum region 0.3–2.5 µm) and strong thermal emission within the atmospheric transparency window (8–13 µm). Mc‐sRCF demonstrates exceptional performance in both solar reflectivity (92.29%) and thermal emissivity (95.34%). (e) Digital photograph of Mc‐sRCF with water contact angle inset (upper right). Our Mc‐sRCF exhibits a white color and hydrophobic character at the macroscopic scale. (f) Schematic illustration of potential environmental stability, including rain resistance, oxidation resistance, wind resistance, and mechanical durability.

The multi‐layer core–shell radiative cooling fabric (Mc‐sRCF) is fabricated via one‐step coaxial electrospinning, which simultaneously constructs the core–shell structure (see Figure S1) and integrates photoluminescent functionality, enhancing both its radiative cooling efficacy and environmental stability (Figure 1a and Figure S2). The inner needle spinning solution is the PEO/Clay‐UP system, and the outer needle spinning solution is the PVDF/ TiO_2_ system (see Supporting Method). While PEO‐based and PVDF‐based coated fibers are exceptional infrared emitters, efficiently dissipating heat absorbed from the ∼6000 K solar source into the ∼3 K cold universe, Clay‐UP and TiO_2_ manage ultraviolet light to facilitate photon conversion and provide UV resistance, respectively. The hierarchical core–shell fiber architecture significantly enhances solar reflectivity and tunable mechanical properties by exploiting multi‐interface reflection and mechanical complementarity, offering a groundbreaking approach to boost both radiative cooling efficiency and structural robustness.

Through the hierarchical structural design of fibers, the toughness of the fiber inner layer and the strength of the outer layer can achieve complementary advantages, enabling the fabric to have outstanding mechanical properties. As shown in Figure 1b, compared to the PEO/Clay‐UP fabric (2.2 MPa, 609.2%) and PVDF/TiO_2_ fabric (3.6 MPa, 152.1%), the Mc‐sRCF demonstrates significantly enhanced tensile strength along with high toughness (8.7 MPa, 336.3%). These remarkable mechanical properties ensure reliable resistance against various natural environmental loads.

Moreover, the TiO_2_ particles encapsulated within the PVDF matrix feature precisely tailored particle sizes to optimize optical properties. Specifically, 60 nm TiO_2_ nanoparticles are selected for their strong ultraviolet absorption, while 500 nm TiO_2_ particles are incorporated due to their exceptional scattering efficiency at the wavelength corresponding to the peak solar irradiance in the visible spectrum. This multiscale TiO_2_ design synergistically endows the fabric with enhanced solar reflectivity and superior UV resistance against photodegradation. As shown in Figure 1c, the Mc‐sRCF fabric embedded with multi‐sized TiO_2_ particles exhibits strong absorption in the UV region. Due to the strong chemical bonding in PVDF, the UV‐absorption properties of TiO_2,_ and the effective ultraviolet reflection at multiple core–shell interfaces, our Mc‐sRCF ultimately demonstrates robust UV resistance.

Benefiting from the intrinsic high infrared emissivity of both PEO and PVDF polymer matrices, coupled with the inherent light‐scattering properties of the fibrous network architecture and TiO_2_ nanoparticles, the multi‐interface reflection effect endowed by the special fiber hierarchical structure, the resulting Mc‐sRCF exhibits exceptional reflectivity/emissivity (92.29%/95.34%) across two crucial spectral windows (visible and mid‐infrared bands), enabling efficient radiative heat rejection to the 3 K cosmic background (Figure 1d). Notably, by converting and re‐emitting ultraviolet light into the visible spectrum, the PL effect contributes additional solar reflectivity gain within specific spectral bands. The synergistic chemical interplay between PEO and Clay‐UP induces dual‐mode photoluminescence (fluorescence (Fl) and phosphorescence (Ph)) in the fabric, facilitating efficient UV–vis photon conversion and ultimately boosting the overall solar reflection performance (the ESR of Mc‐sRCF reaches 101.1%). The high emission efficiency and excellent reflective properties of our Mc‐sRCF highlight its remarkable potential for radiative cooling applications. Ultimately, our Mc‐sRCF displays a macroscopically white appearance, offering exceptional radiative cooling efficiency and outstanding outdoor durability (Figure 1e). Its comprehensive environmental stability, including oxidation resistance, intrinsic hydrophobicity (a water contact angle of 130.7°, see Figure 1e), and mechanical robustness, provides holistic protection against diverse climatic stressors such as extreme heat, rainfall, and high‐speed winds (Figure 1f).

### 2.2 Photoluminescence of Radiative Cooling Mc‐sRCF

**FIGURE 2:**
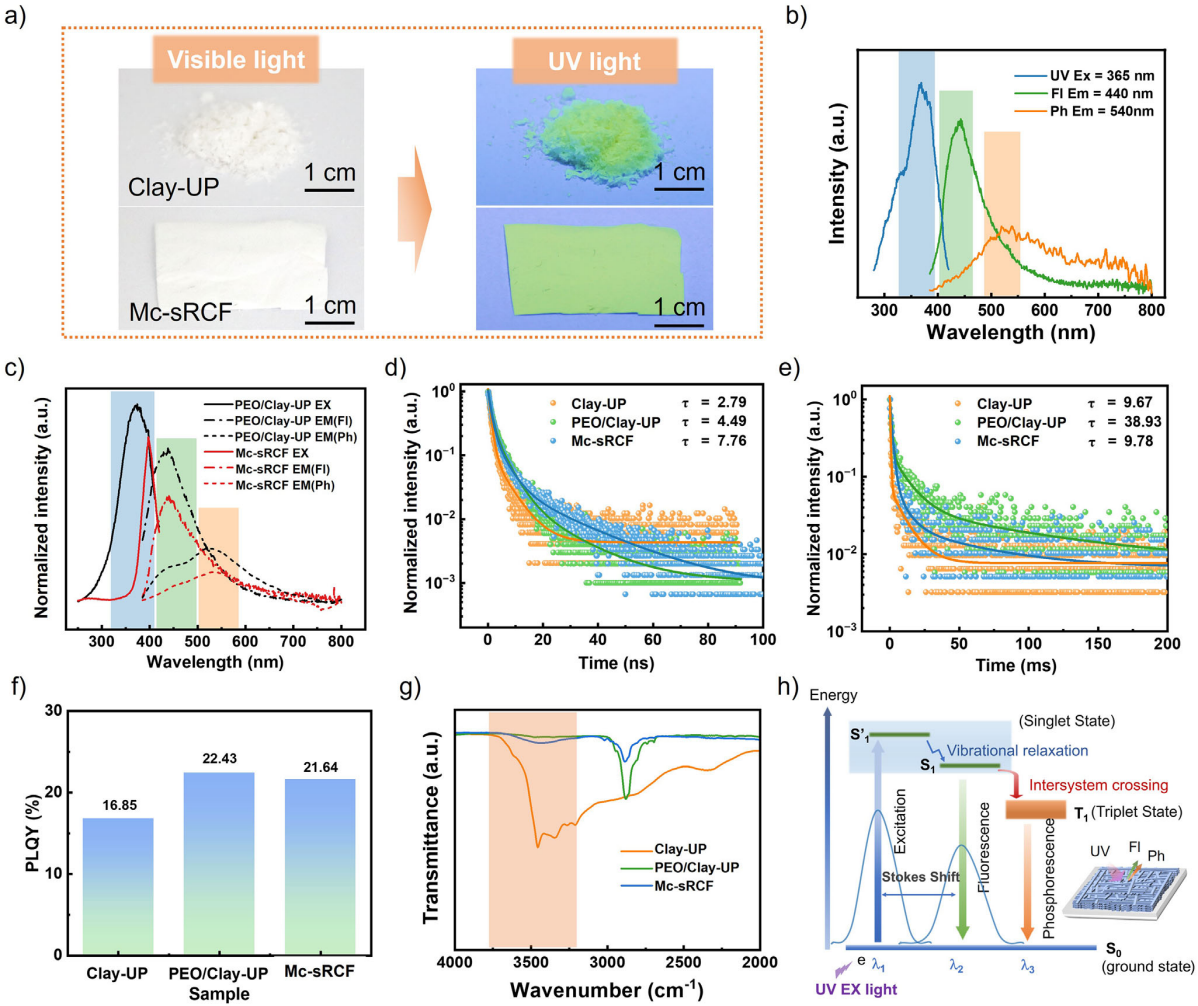
Photoluminescence characteristics and underlying mechanisms of the Mc‐sRCF. (a) Digital photographs of Clay‐UP filler and Mc‐sRCF under visible light and UV light (365 nm), demonstrating their photoluminescence. (b) Fluorescence (440 nm) and phosphorescence (540 nm) emission spectra of Clay‐UP under 365 nm UV excitation. The result indicates that the dual‐mode photoluminescence (fluorescence and phosphorescence) of Clay‐UP. (c) Fluorescence and phosphorescence emission spectra of PEO/Clay‐UP fabric and Mc‐sRCF. It confirms the inherited nature of the photoluminescence in the Mc‐sRCF material. (d) Fluorescence decay lifetimes and (e) Phosphorescence decay lifetimes of Clay‐UP, PEO/Clay‐UP fabric, and Mc‐sRCF. Our Mc‐sRCF significantly extends the luminescence lifetime (fluorescence: 7.76 ns; phosphorescence: 9.78 ms). (f) Absolute photoluminescence quantum yields (PLQY) of different samples. The resulting PLQY of Mc‐sRCF increases to 21.64%, indicating a marked enhancement. (g) FTIR spectra of different samples. The result reveals that the hydrogen‐bonding network is undergoing changes and improves the photon‐conversion efficiency. (h) Schematic illustration of the photoluminescent mechanism in Mc‐sRCF (The symbols S_0_, S_1_, and T_1_ refer to the ground state, the first excited singlet state, and the first excited triplet state, respectively).

Photoluminescence converts ultraviolet photons into visible wavelengths to enable enhancement of radiative cooling through enhancing solar reflectivity, which is the key application proposed in this work. As shown in Figure 2a, under irradiation (365 nm) with a 500 W high‐pressure UV mercury lamp, the Mc‐sRCF emits green light. To verify the photoluminescence origin, we systematically investigate the excitation and emission spectra of Clay‐UP, PEO, PEO/Clay‐UP, and Mc‐sRCF (Figure 2b,c, and Figure S3). It is found that the PEO fabric exhibited no emission upon UV excitation (see Figure S3), whereas the other materials containing Clay‐UP show varying degrees of fluorescence and phosphorescence emission. Specifically, the pristine Clay‐UP spectrum (Figure 2b) exhibits an excitation peak at 375 nm, with dual emission bands centered at 440 nm (fluorescence) and 540 nm (phosphorescence), the latter showing attenuated intensity. The Mc‐sRCF and PEO/Clay‐UP demonstrate comparable photoluminescent behavior to the Clay‐UP, with closely matched excitation‐emission profiles. Notably, the overall photoluminescent property of Mc‐sRCF remains intact (Figure 2c), despite a slight decrease in its fluorescence‐phosphorescence intensity due to the newly introduced shell, when compared to the PEO/Clay‐UP fabric. All results confirm the inherited photoluminescent behavior from the Clay‐UP filler rather than from PEO, and the formation of the shell does not compromise the luminescent capacity of the core. As shown in Figure 2d,e, PEO/Clay‐UP fabric and Mc‐sRCF exhibit prolonged fluorescence and phosphorescence lifetimes (fluorescence: 4.49/7.76 ns; phosphorescence: 38.93/9.78 ms). The persistent phosphorescence originates from photon recycling via extended optical path lengths [36] and the extended fluorescence lifetime of Mc‐sRCF results from confinement‐induced stabilization by the protective shell. Prolonged photoluminescence allows more time for energy transfer and relaxation processes, thereby further suppressing non‐radiative transitions and reducing heat generation.

To evaluate the photonic contribution to radiative cooling, we further quantify the solar spectrum photon conversion efficiency via absolute photoluminescence quantum yield (PLQY) measurements (Figure 2f). Both PEO/Clay‐UP fabric and Mc‐sRCF maintain competitive peak PLQY values, at 22.43% and 21.64% respectively, which significantly exceed the PLQY of the original Clay‐UP (16.85%). The reason is that, for Clay‐UP, it has an extensive hydrogen‐bonding network (Figure 2g), which facilitates efficient intramolecular charge transfer, thereby inducing the observed photoluminescence. Upon intimate contact between the Clay and PEO within the PEO/Clay‐Up fabric and Mc‐sRCF, the nucleophilic affinity between PEO ether oxygens and Clay hydroxyl groups improves the photon‐conversion efficiency [37]. In addition, the mixed single‐layer structure fabric, serving as a control, shows a slightly elevated fluorescence emissivity and PLQY (21.82%) relative to the Mc‐sRCF fabric (yet inferior to the PEO/Clay‐UP fabric). This increase is attributed to the exposure of the highly photoluminescent PEO/Clay‐UP components originally encapsulated within the core of the core–shell structured fibers (Figure S4).

The schematic concisely illustrates the fundamental photoluminescence pathways in molecular systems (Figure 2h). Upon UV‐visible photoexcitation, the electrons in molecules of Clay‐UP and PEO/ Clay‐UP promote from the singlet ground state (S_0_) to an upper vibronic level of an excited singlet state (S_n_) (n ≥ 1). This electronic transition is primarily driven by a cross‐component hydrogen‐bonding network at the interfaces. Ultrafast vibrational relaxation subsequently funnels the population to the lowest vibrational level (v = 0) of the lowest excited singlet state (S_1_). From S_1_, two competing deactivation channels emerge: (i) radiative decay, giving rise to Stokes‐shifted fluorescence back to S_0_; and (ii) non‐radiative intersystem crossing to the lowest triplet state (T_1_), which ultimately yields phosphorescence within Clay‐UP and the fabrics. Owing to these two channels, our Mc‐sRCF exhibits spontaneous photoluminescence, which converts a portion of absorbed sunlight below 400 nm into visible‐region fluorescence and phosphorescence emissions, leading to enhancing the ESR and reducing overall solar absorption (see Note S3.1).

### 2.3 Solar Spectrum Optimization and Radiative Cooling through Hierarchical Core–Shell Fiber architectures and Precision Dimensional Control

**FIGURE 3:**
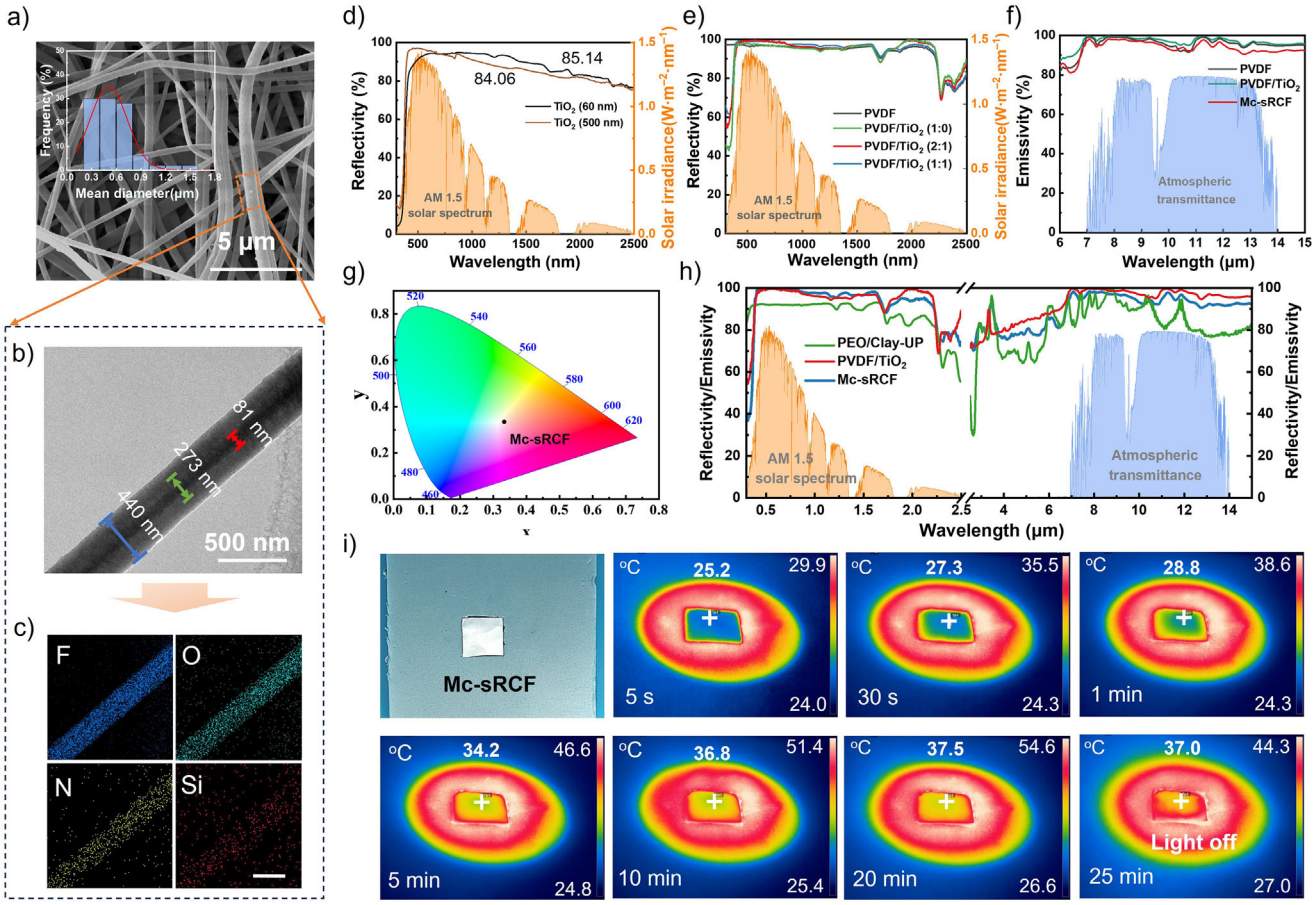
Spectral optimization and radiative cooling properties of Mc‐sRCF. (a) SEM micrograph of Mc‐sRCF with corresponding fiber diameter distribution. Statistical analysis reveals an average fiber diameter of 500 nm. (b) High‐resolution TEM image of an individual Mc‐sRCF fiber. It confirms an advanced tri‐layer core–shell architecture of Mc‐sRCF, comprising a Clay core, a PEO intermediate layer, and a PVDF outer shell. (The corresponding fiber diameters are measured at 440 nm for Mc‐sRCF, 273 nm for PEO/Clay‐UP, and 81 nm for the Clay). (c) Elemental mapping visualizes the elemental distribution within the three‐layer architecture of Mc‐sRCF fiber. (d) Solar reflectivity spectra of TiO_2_ particles at different scales (60 nm versus 500 nm). The results highlight their respective advantages across the solar spectrum. (e) Solar reflectivity spectra of PVDF fabrics incorporating TiO_2_ particles with varying size ratios (60: 500 nm). PVDF/TiO_2_ (2:1) shows the highest solar reflectivity among the hybrid PVDF/TiO_2_ fabrics, achieving an average solar reflectivity of 92.05%. (f) Mid‐infrared emission spectra of PVDF fabric, PVDF/TiO_2_ fabric, and Mc‐sRCF. It reveals the optimized infrared emission capability of Mc‐sRCF. (g) CIE chromaticity coordinates demonstrating the whiteness of Mc‐sRCF. (h) Broadband solar spectral response of PEO/Clay‐UP fabric, PVDF/TiO_2_ fabric, and Mc‐sRCF, including solar reflectivity (0.3–2.5 µm) and thermal emissivity (8–13 µm). It showcases the optimized solar reflection (92.29%) and infrared emission (95.34%) capabilities of Mc‐sRCF. (i) Time‐resolved infrared thermography of Mc‐sRCF under 1‐sun illumination (1000 W·m^−^^2^) captures its dynamic temperature evolution over time. At the steady state (after 20 min), Mc‐sRCF maintains a temperature 17.1 °C lower than the ambient foam, confirming its highly efficient radiative cooling performance.

The microstructure design and precise dimensional control of the fibers are important for fundamentally optimizing solar spectral management. The morphology and diameter distribution of the Mc‐sRCF fibers are presented in Figure 3a. Utilizing coaxial electrospinning, the fiber diameter is precisely engineered to 500 nm as designed (exceeding those of PVDF/TiO_2_ fibers due to the formation of a core–shell structure, see Figure S5), which precisely corresponds to the peak wavelength of solar irradiance to effectively enhance solar reflectivity. This demonstrates the success of our structure design in regulating the fiber diameter. Additionally, Mc‐sRCF possesses both a large specific surface area (2.49 m^2^/g) and a small average pore size (14 nm), which collectively contribute to enhanced solar reflectivity (see Note S3.2, Table S1, and Figure S6). More interestingly, the transmission morphology (TEM) and characteristic elemental mapping of individual fibers (Figure 3b,c) unequivocally confirm an advanced tri‐layer core–shell architecture of Mc‐sRCF, comprising a Clay core, a PEO intermediate layer, and a PVDF outer shell, in contrast to the two‐layer configuration without a Clay core (Figure S7). This distinctive multi‐layer architecture is expected to significantly enhance solar reflectivity by promoting multiple internal reflections of sunlight.

According to Rayleigh scattering theory [38], small‐sized nanoparticles exhibit broad‐spectrum reflection at wavelengths exceeding 10 times their diameter. In contrast, particles with diameters comparable to the incident wavelength can scatter light of the corresponding wavelength, based on Mie scattering theory [39]. Therefore, a dual‐size strategy employing TiO_2_ particles of 60 and 500 nm is adopted to maximize solar reflectivity by capitalizing on their respective advantages. As shown in Figure 3d, the 60 nm TiO_2_ particles demonstrate high reflectivity across the entire solar radiation region. Meanwhile, the 500 nm TiO_2_ particles achieve targeted reflection specifically around the 500 nm wavelength (corresponding to peak solar irradiance) (Figure 3d), with an exceptional reflectivity reaching 97.09%. By optimizing the mass ratio between the two particles, the hybrid with a 2:1 ratio of 60 and 500 nm TiO_2_ particles has the highest solar reflectivity, achieving an average solar reflectivity of 91.95% (Figure 3e). Throughout the following text, the designation “PVDF/TiO_2_” specifically denotes the fabric embedded with TiO_2_ nanoparticles at a mass ratio of 2:1 (60 and 500 nm particle sizes).

The fabrics are subjected to elemental composition analysis coupled with a thorough assessment of their infrared absorption behavior across the “atmospheric window” region (see Figure S8a,b). Both the stretching vibration of the C─F bonds from the PVDF component and the stretching vibration of the C─O─C bonds from the PEO component exhibit distinct absorption bands in this window. Combined with the phonon resonance effect of TiO_2_, [40] Mc‐sRCF exhibits a high mid‐infrared emissivity of 95.34% (Figure 3f). The CIE chromaticity diagram demonstrates the exceptional whiteness of the Mc‐sRCF, visually confirming its high solar reflectivity (Figure 3g). Ultimately, our Mc‐sRCF achieves ultrahigh average solar reflectivity (ASR, 92.29%), particularly in the high solar thermal radiation band of 0.5–1.8 µm (97.04%), and average mid‐infrared emissivity of 95.34% (Figure 3h). Interestingly, a control experiment revealed that the single‐layer, uniaxial electrospun fibers prepared via the blending method exhibit a solar reflectance of 88.19% and an infrared emittance of 94.92%, both of which are notably lower than those of our Mc‐sRCF fabric (see Figure S9). This stark contrast provides compelling evidence that constructing multilayer core–shell structured fibers via coaxial electrospinning offers a significant advantage in enhancing the spectral regulation capability of the material. Notably, the contribution of photoluminescence to the solar reflectivity is quantitatively evaluated. The Mc‐sRCF achieves a remarkable average effective solar reflectivity (AESR) of 93.45%, with a peak exceeding 100% (101.1%) in the specific spectral band, providing definitive evidence for photon recycling as theoretically established by calorimetry (Note S3.1). The combination of high solar reflectivity and strong thermal emissivity collectively forms the physical foundation for its exceptional radiative cooling performance.

Figure 3i presents the time‐dependent infrared thermal imaging of Mc‐sRCF under a 1000 W·m^−^^2^ solar simulator. The temperature of Mc‐sRCF shows an initial rapid increase of 9.0 °C within the first 5 min, compared to a much sharper rise of 16.7 °C for the polystyrene foam. Then, the heating rate slows, with an additional gain of 3.3 °C for the fabric versus 4.8 °C for the foam over the next 5 min. The temperatures eventually stabilize at 37.5 °C for the fabric and 54.6 °C for the foam after 20 min of irradiation (see Figure 3i). Throughout the exposure period, Mc‐sRCF consistently maintains a lower temperature than the surrounding polystyrene foam, demonstrating its excellent solar reflectance performance, which effectively reduces heat accumulation. Removing the light source at 25 min causes the foam temperature to drop immediately from 54.6 °C to 44.3 °C. In contrast, Mc‐sRCF shows minimal cooling with a decrease of only 0.5 °C, indicating its exceptional thermal insulation properties under ambient conditions. The exceptional thermal insulation is crucial for the practical application of radiative cooling, as it effectively mitigates environmental heat ingress and enhances the net cooling power. Similarly, in laboratory radiative cooling tests, the Mc‐sRCF achieves an average temperature reduction that is 9.2°C, 2.8°C, and 1.6°C lower than that of the cavity, PEO/Clay‐UP fabric, and PVDF/TiO_2_ fabric, respectively (Figure S10), demonstrating its superior cooling performance among comparable fabrics. Furthermore, under a solar irradiance of 1275 W·m^−^^2^ over 25 min, the temperature of the Mc‐sRCF increases gradually to 44.7 °C, which is 4.6 °C and 2.4 °C lower than that of the PEO/Clay‐UP fabric (49.3 °C) and the PVDF/TiO_2_ fabric (47.1 °C), respectively (Figure S11), demonstrating its excellent cooling performance even under high solar intensity.

### 2.4 Exceptional UV‐Degradation Stability and Mechanical Durability in Mc‐sRCF

Resistance to UV aging remains a critical challenge for organic radiative coolers, as it is essential for ensuring their long‐term stability in outdoor applications. In contrast to conventional organic UV stabilizers, which degrade and lose effectiveness over time, inorganic UV absorbers exhibit superior weatherability. TiO_2_, with its unique optical properties and chemical stability, not only efficiently absorbs UV radiation (particularly in the UV‐A and UV‐B bands, through its wide bandgap structure), but also enhances UV shielding via light‐scattering effects at the nanoscale, endowing it with tremendous potential for outdoor applications in radiative coolers. Therefore, the use of dual‐sized TiO_2_ particles is highly strategic, enabling dual UV shielding by leveraging the absorption of small particles and the reflection of large ones.

**FIGURE 4:**
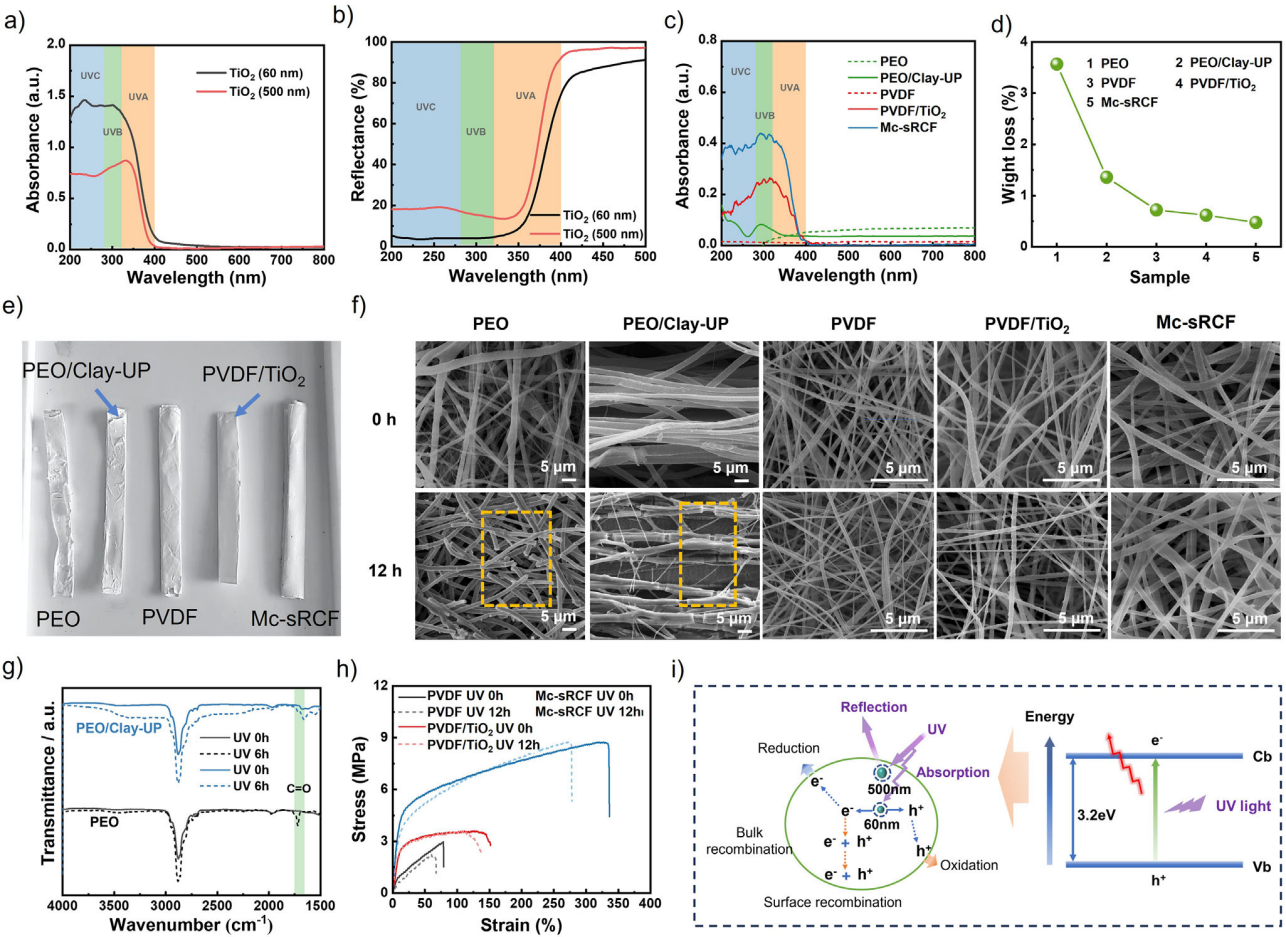
UV resistance property of Mc‐sRCF. (a) UV–vis absorption spectra of 60 and 500 nm TiO_2_ nanoparticles, revealing the pronounced absorption of the smaller particles (60 nm). (b) The UV reflectance spectra of 60 and 500 nm TiO_2_ particles reveal that the larger particles (500 nm) exhibit enhanced reflectivity. For our Mc‐sRCF, a binary particle size system may create a synergistic effect to effectively attenuate incoming UV radiation. (c) The comparative UV absorption profiles of PEO fabric, PEO/Clay‐UP fabric, PVDF fabric, PVDF/TiO_2_ fabric, and Mc‐sRCF. It demonstrates the synergistically enhanced absorption of Mc‐sRCF. (d) Mass change of PEO fabric, PEO/Clay‐UP fabric, PVDF fabric, PVDF/TiO_2_ fabric, and Mc‐sRCF following 12 h of UV high‐pressure mercury lamp (500 W) irradiation. Our Mc‐sRCF exhibits the lowest mass loss (<0.5%). (e) Macroscopic digital photographs of the PEO fabric, PEO/Clay‐UP fabric, PVDF fabric, PVDF/TiO_2_ fabric, and Mc‐sRCF after 12 h of UV high‐pressure mercury lamp (500 W) irradiation. Mc‐sRCF shows no signs of morphological degradation, showcasing its superior UV stability. (f) SEM morphologies of PEO fabric, PEO/Clay‐UP fabric, PVDF fabric, PVDF/TiO_2_ fabric, and Mc‐sRCF, before and after 12 h of UV high‐pressure mercury lamp (500 W) irradiation. Our Mc‐sRCF fibers show no signs of fracture. (g) FTIR spectra of PEO and PEO/Clay‐UP fabrics before and after 6 h of UV exposure. The result indicates that the photooxidative cleavage of their low‐bond‐energy C─O─C ether linkages. (h) Stress‐strain curves of PVDF fabric, PVDF/TiO_2_ fabric, and Mc‐sRCF before and after 12 h of UV irradiation, demonstrating the superior mechanical strength retention of Mc‐sRCF (tensile strength: 8.7 MPa; elongation at Break: 336.3%). (i) Proposed mechanism illustrating the UV‐resistant behavior of Mc‐sRCF. While 60 nm TiO_2_ nanoparticles absorb UV radiation and dissipate the energy through bulk recombination, surface recombination, and redox reactions of the electron‐hole pairs, the 500 nm counterparts primarily shield the substrate by physically reflecting UV light, thus preventing its penetration and damage.

Consistent with size‐dependent optical properties, the 60 nm TiO_2_ nanoparticles demonstrate significantly enhanced UV absorbance (up to 1.46 at maximum) across the entire UV spectrum compared to their 500 nm counterparts, attributed to a pronounced nano‐size effect, which acts to amplify UV absorption through a combination of increased specific surface area and quantum size‐mediated bandgap modulation. Meanwhile, the distinct blue shift observed in the 60 nm TiO_2_ serves as direct evidence of its enhanced short‐wave ultraviolet (UVC) absorption and wider UV protection range compared to larger particles (Figure 4a). Furthermore, the 500 nm TiO_2_ particles exhibit substantial UV reflectivity (see Figure 4b), which, when combined with the absorption by smaller particles, creates a synergistic effect that thus effectively attenuates incoming UV radiation. In the binary particle size system, the PVDF/TiO_2_ fabric demonstrates stronger UV absorbance compared to the pure PVDF fabric (Figure S12). Fortunately, compared with PEO/Clay‐UP fabric and PVDF/TiO_2_ fabric, the Mc‐sRCF with a hierarchical core–shell structure exhibits higher UV absorbance (0.44, see Figure 4c). This is mainly attributed to the synergistic UV absorption of TiO_2_ and Clay‐UP, rather than the pure PEO or PVDF matrix. While Clay‐UP absorbs UV light only to re‐emit it at longer wavelengths for photoluminescence, TiO_2_ provides the substantive UV protection.

The UV resistance of the fabrics is evaluated by examining changes in their macroscopic and microscopic morphology as well as mass variation. Weight analysis confirms distinct differences in UV stability among the fabrics (Figure 4d). While the PVDF‐based fabrics show excellent UV stability with mass loss <1%, PEO and PEO/Clay‐UP fabrics have significantly higher degradation rates (especially pure PEO with 3.57% mass loss). Remarkably, Mc‐sRCF exhibits nearly negligible mass loss (<0.5%), clearly demonstrating its outstanding UV shielding effect. Morphological observations indicated that, after 6 h of UV exposure, the PEO fabric begins to exhibit fiber breakage, while no observable morphological changes are detected in the other fabrics (Figure S13). After 12 h of continuous UV irradiation, PEO and PEO/Clay‐UP fabrics suffer obvious degradation (Figure 4e,f) due to the photooxidative cleavage of their low‐bond‐energy C ‐ O ‐ C ether linkages (Figure 4g). PEO fabrics shrank with most ultrafine fibers broken, while PEO/Clay‐UP fabrics have impaired structural integrity, though fibers are not completely broken due to protective Clay in the core, which provided physical UV isolation. However, PVDF, PVDF/TiO_2_, and the hierarchical Mc‐sRCF fabrics maintain intact morphology without detectable changes (Figure 4e,f). This is attributed to the strong C ‐ F bond energy and UV absorption of TiO_2_ in PVDF‐based systems, which prevents polymer matrix degradation. These results suggest that the superior UV protection performance of Mc‐sRCF originates from its PVDF/TiO_2_ shell shielding the PEO/Clay core from photodegradation, as well as efficient UV reflection achieved at the multiple core–shell interfaces.

In contrast to the PEO (1.0 MPa, 122.7%), PEO/Clay‐UP (2.2 MPa, 609.2%), PVDF (3.0 MPa, 78.3%), and the PVDF/TiO_2_ (3.6 MPa, 152.1%) fabrics, our Mc‐sRCF exhibits significantly enhanced tensile strength and high toughness (8.7 MPa, 336.3%) (Figure 1b and Figure S14). Compared to the pure PVDF fabric, the enhanced strength of the PVDF/TiO_2_ fabric is primarily attributed to the uniform dispersion and strong interfacial bonding of the TiO_2_ nanoparticles. These factors facilitate effective stress transfer, promote the formation of a finer crystalline structure, and induce crack deflection, collectively contributing to the improved mechanical performance. For Mc‐sRCF, its superior strength primarily originates from its hierarchical fibrous structure and densely interwoven network with PVDF encapsulation, while its high toughness is attributed to the flexible PEO molecular chains. Even after 12 h of UV irradiation, Mc‐sRCF also retains optimal mechanical stability (Figure 4h). Specifically, the morphological degradation of PEO and PEO/Clay‐UP fabrics precludes the detection of their subsequent tensile behavior. For the PVDF fabric, a significant reduction in tensile strength (26.4%) is observed after UV irradiation. In contrast, the PVDF/TiO_2_ fabric (<0.1%) and Mc‐sRCF (<0.3%) show no significant loss in mechanical strength after UV exposure due to the UV‐shielding effect of TiO_2_. Notably, even when subjected to 100 loading‐unloading cycles at deformation strains (10% and 20%) beyond their yield points, the UV‐treated Mc‐sRCF maintains excellent mechanical stability, exhibiting a tensile strength retention of over 80% (Figure S15). These results confirm our Mc‐sRCF superior mechanical durability.

Figure 4i demonstrates the UV‐shielding mechanism of dual‐sized TiO_2_. The 60 nm TiO_2_ nanoparticles exhibit excellent UV resistance due to their 3.2 eV bandgap, which efficiently absorbs UV light and generates electron‐hole pairs. Bulk recombination dissipates energy as heat, while surface recombination allows holes (h^+^) to react with adsorbed species, passivating surface defects. The small particle size enhances charge separation and minimizes recombination losses, ensuring our Mc‐sRCF long‐term stability under UV exposure. In parallel, the 500 nm TiO_2_ nanoparticles reflect UV‐band radiation, reducing UV penetration. Through this synergistic action, the dual‐sized TiO_2_ particles collectively shield the matrix from UV damage, thereby providing robust anti‐UV functionality.

### 2.5 Comprehensive Environmental Stability of Mc‐sRCF

**FIGURE 5:**
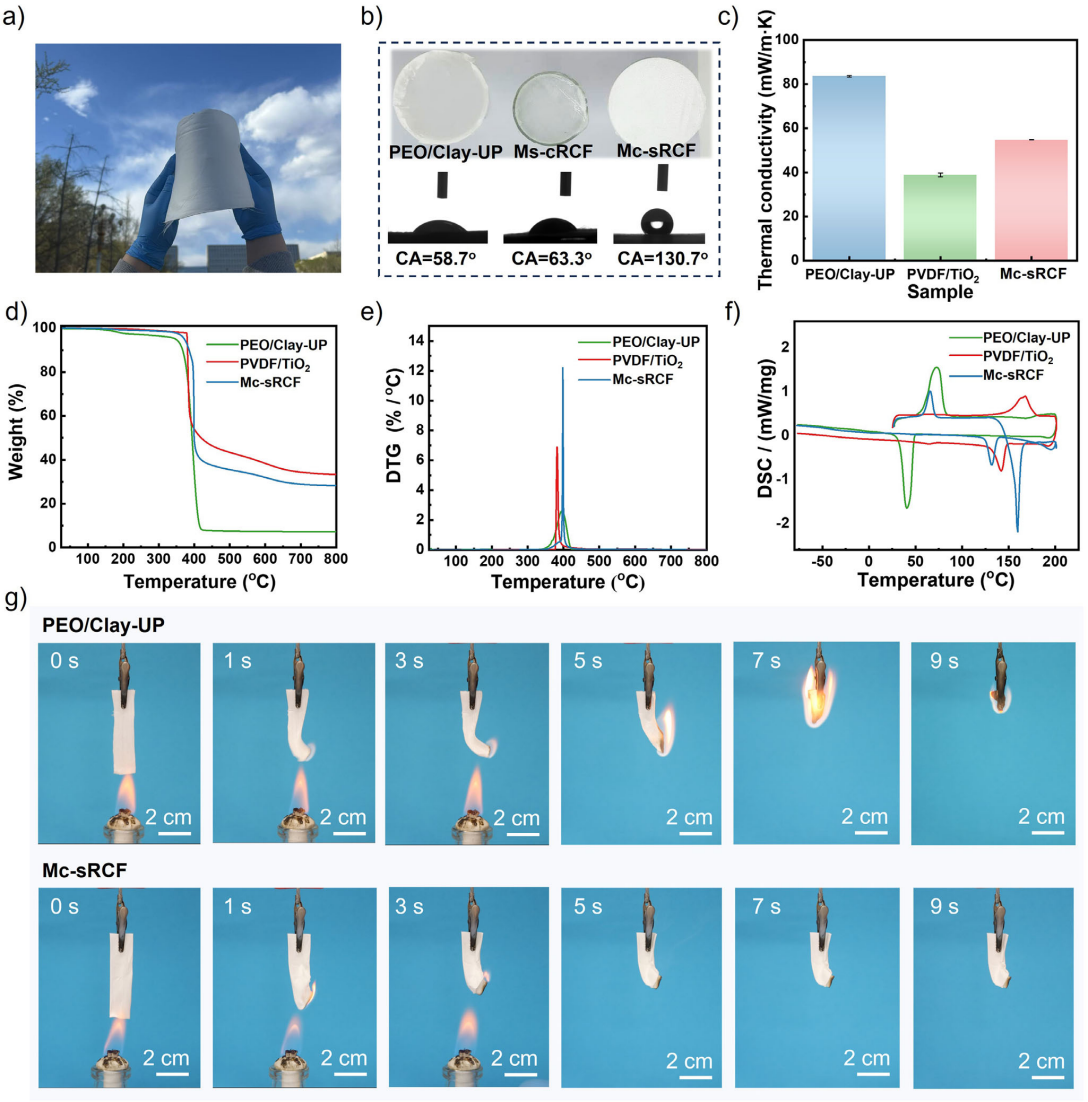
Environmental stability of Mc‐sRCF. (a) Flexibility demonstration of Mc‐sRCF under Category 6 gale‐force wind resistance conditions. (b) Water corrosion resistance (digital photographs) and comparative contact angle measurements of the PEO/Clay‐UP fabric (58.7°), Ms‐cRCF (fabric with PVDF/TiO_2_ as the core and PEO/Clay‐UP as the shell, 63.3°), and Mc‐sRCF (130.7°). The results highlight the superior hydrophobicity of the Mc‐sRCF. (c) Thermal conductivity coefficients of PEO/Clay‐UP fabric, PVDF/TiO_2_ fabric, and Mc‐sRCF. The thermal conductivity of Mc‐sRCF is as low as 52 mW/m·K, indicating a highly effective thermal insulation capability. (d–f) Thermal stability analysis, including TGA, DTG, and DSC thermograms. The exceptional thermal stability of Mc‐sRCF underpins its effective thermal protection capability. (g) Time‐dependent combustion behavior of PEO/Clay‐UP fabric and Mc‐sRCF. Mc‐sRCF extinguishes flame entirely after the flame source is removed at 5 s, demonstrating its self‐extinguishing properties and highlighting its excellent flame‐retardant performance.

The environmental stability of radiative cooling fabrics is a pivotal criterion for their practical deployment in outdoor settings [41]. As demonstrated in Figure 5a, the Mc‐sRCF exhibits remarkable mechanical resilience, maintaining exceptional flexibility and impact resistance even under Category 6 gale‐force wind, confirming its structural robustness in extreme weather conditions. Beyond wind resistance, the Mc‐sRCF demonstrates superior durability against environmental erosion. After repeated abrasion on sandpaper, PEO/Clay‐UP and PVDF/TiO_2_ fabrics show weight reductions of 1.89% and 22.58%, respectively, while Mc‐sRCF maintains structural integrity with merely superficial fuzzing, and no measurable mass loss (Figure S16). Notably, the PVDF/TiO_2_ fabric suffers complete surface failure, attributable to the structural fragility of its ultrafine short fibers. Compared to the PVDF/TiO_2_ fabric, the superior abrasion resistance of our Mc‐sRCF fabric originates from the robust support of its long‐fiber core (PEO/Clay‐UP) and more stable hierarchical core–shell structure, which collectively contribute to its enhanced mechanical endurance.

After two months of outdoor degradation testing, the PEO/Clay‐UP fabric develops visible cracks as early as day 30, whereas our Mc‐sRCF shows no significant signs of degradation (Figure S17). Moreover, Mc‐sRCF features high hydrophobicity (CA = 130.7°) and anti‐fouling properties, attributed to the hydrophobic PVDF/TiO_2_ shell layer (Figure 5b, Figure S18 and Movies S1, S2). This high hydrophobicity also arises from its finer micro‐nano surface morphology and smaller average pore size, which enhances capillary resistance and reduces surface energy. These characteristics effectively minimize dust accumulation and mitigate damage to the Mc‐sRCF caused by rainwater exposure. Furthermore, the Mc‐sRCF exhibits ultralow thermal conductivity (52 mW/m·K, see Figure 5c), endowing it with an exceptional thermal insulation property that effectively suppresses heat transfer. This unique characteristic makes it particularly valuable for protecting underlying structures from thermal hazards, especially in radiative cooling systems for buildings and telecommunications equipment.

The thermal stability of PEO fabric, PEO/Clay‐UP fabric, PVDF fabric, PVDF/TiO_2_ fabric, and Mc‐sRCF is systematically investigated through thermogravimetric (TG, 30–800 °C), derivative thermogravimetry (DTG), and differential scanning calorimetry (DSC, ‐110–200 °C) analyses (Figure 5d–f, and Figure S19). PEO/Clay‐UP exhibits a two‐stage degradation profile, with initial weight loss at ∼170 °C corresponding to volatile release (urea phosphate and water) followed by major polymer backbone cleavage at ∼350 °C (as evidenced by a relatively broad and shallow peak in the DTG curve, see Figure 5e), ultimately yielding only 8% residue at 800 °C. In contrast, PVDF/TiO_2_ and Mc‐sRCF demonstrate significantly enhanced thermal resistance with decomposition onset temperatures (T_o__n__e__t_) of ∼380 °C and 350 °C, respectively. The DTG curves of both PVDF/TiO_2_ and Mc‐sRCF demonstrate sharper and more intense decomposition peaks (Figure 5e). These are attributed to the strong C ‐ F bonds of PVDF and thermal barrier effect of TiO_2_, resulting in substantially higher char residues of 33% and 28% at 800 °C. Notably, Mc‐sRCF displays a pronounced positive temperature shift in its decomposition peak, demonstrating enhanced thermal stability relative to the other composites.

The DSC analysis further elucidates the thermal behavior of these composites (Figure 5f). A sharp melting endotherm at 70 °C is observed for PEO/Clay‐UP, characteristic of crystalline PEO domain melting. In contrast, PVDF/TiO_2_ exhibits a distinct melting transition at 160 °C, corresponding to the α‐crystal melting of PVDF, with this melting temperature being significantly higher than that of PEO/Clay‐UP. For Mc‐sRCF, the DSC profile is far more stable across the entire temperature range (from −50 °C to 200 °C), demonstrating that Mc‐sRCF undergoes negligible thermal decomposition or phase transition. It seems that the core–shell architecture of Mc‐sRCF endows exceptional thermal stability, as manifested by its elevated decomposition temperature and preserved structural integrity, demonstrating the effective thermal shielding capability of the protective shell layer.

We further characterize the flame retardancy of these fabrics, as presented in Figure 5g and Figure S20. During combustion testing, all three fabrics (PEO/Clay‐UP, PVDF/TiO_2_, and Mc‐sRCF) exhibit initial deformation, shrinkage, and partial ablation within the first 3 s. However, PVDF/TiO_2_ fabric and Mc‐sRCF demonstrate significantly smaller flame areas compared to PEO/Clay‐UP. After removing the flame source at 5 s, PEO/Clay‐UP continues to burn until 9 s, almost completely charred (Movie S3). In contrast, PVDF/TiO_2_ fabric and Mc‐sRCF extinguish their flames entirely after the flame source was removed at 5 s, demonstrating their self‐extinguishing properties and highlighting their excellent flame‐retardant performance (Movie S4). The remarkable environmental stability of Mc‐sRCF significantly advances its potential for real‐world deployment.

Notably, in comparison with the original Mc‐sRCF fabric with a solar reflectance of 92.29% and infrared emission of 95.34%, the Mc‐sRCF fabric retains a solar reflectance of 91.66% and an infrared emission of 95.12% after sequential exposure to UV radiation, water, and acid erosion. These minimal changes fall within the measurement error range, further underscoring its excellent environmental stability (Figure S21).

### 2.6 Actual Outdoor Cooling Performance Evaluation of Mc‐sRCF

**FIGURE 6:**
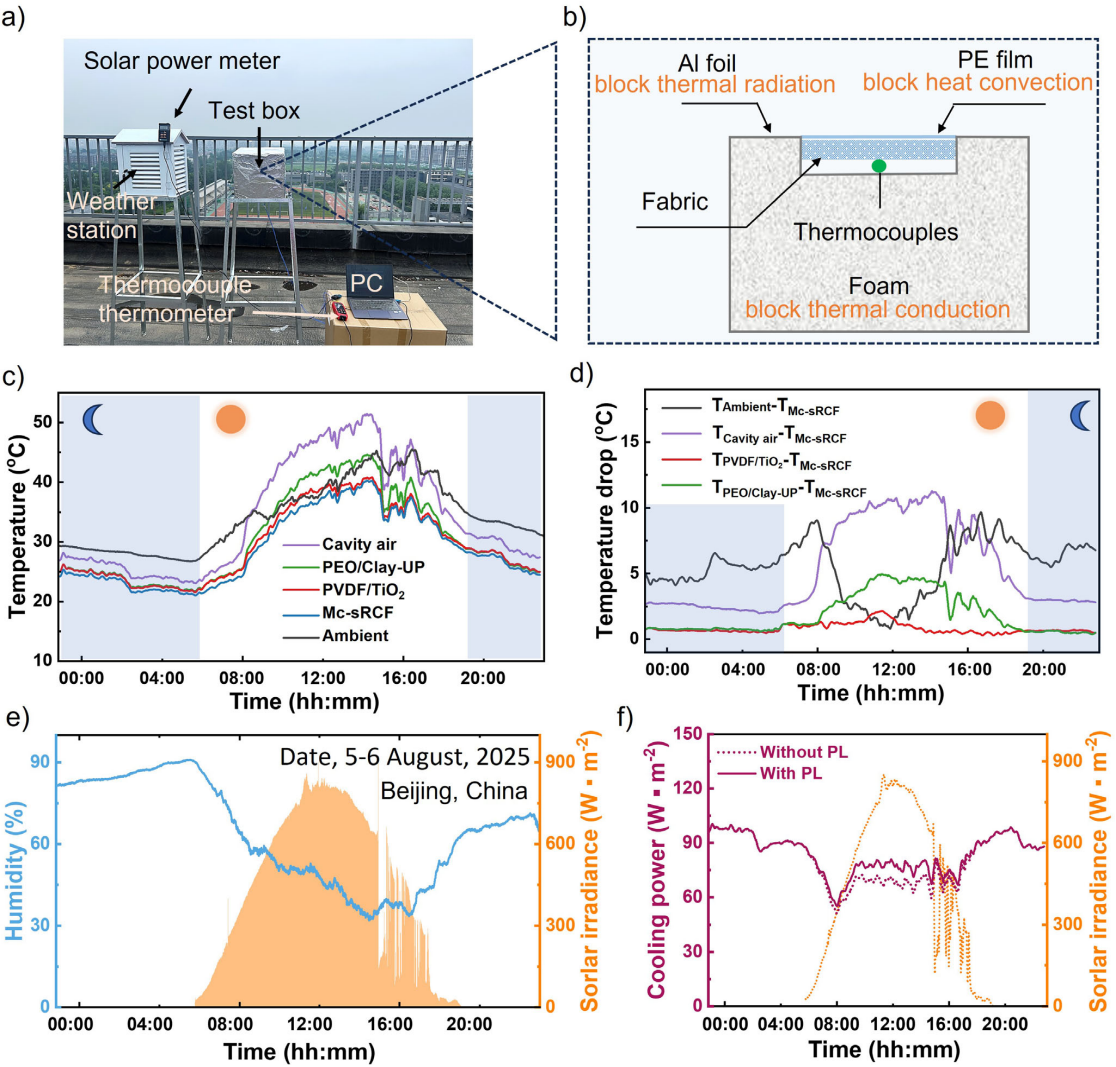
Actual outdoor cooling performance evaluation. (a) Digital photograph of the radiative cooling test setup, including a solar power meter, weather station, thermocouple thermometer, test box, and a data acquisition PC. (b) Cross‐sectional schematic diagram of the test box in front perspective view. The test foam box dimensions are 30 cm × 30 cm× 30 cm, and the embedded sample dimensions are 8 cm × 8 cm× 0.06 cm. (c) 24 h diurnal temperature profiles of tested fabrics from August 5–6, 2025. (d) Temperature drops comparison between ambient, cavity air, PVDF/TiO_2_ fabric, and PEO/Clay‐UP fabric with Mc‐sRCF. Among the tested materials, Mc‐sRCF demonstrated superior passive radiative cooling performance, achieving the largest temperature drop with average reductions of 5.2 °C (nighttime) and 3.9 °C (daytime) below ambient temperature. (e) Environmental humidity and solar irradiance parameters recorded during August 5–6, 2025. (f) Cooling power of Mc‐sRCF with and without photoluminescence (PL) effects. Mc‐sRCF exhibits a total cooling power of 83.78 W·m^−^^2^, of which the photoluminescence effect accounts for 2.88 W·m^−^^2^.

The outdoor radiative cooling performance of Mc‐sRCF is systematically evaluated through field experiments conducted in Beijing, China (40.1512°N, 116.2793°W, altitude: 46 m) during August 5–6, 2025. As illustrated in Figure 6a, the experimental setup consists of a solar power meter (for measuring solar irradiance), a weather station (for monitoring ambient temperature and humidity), a thermocouple thermometer (for recording surface temperatures of fabric samples), a custom‐designed test box, and a data acquisition PC. Figure 6b presents a schematic cross‐section of the test box, detailing the configuration of fabric samples and key components. The test box is designed with multiple thermal insulation features: an outer aluminum foil layer to block thermal radiation, a polyethylene film cover minimizing convective heat transfer, and a high‐density polystyrene foam base effectively suppressing conductive heat exchange from the ground and surrounding environment. Thermocouple sensors are strategically positioned beneath the fabric samples to monitor temperature variations with high temporal resolution continuously.

To demonstrate the cooling performance of Mc‐sRCF, we conduct comparative experiments measuring four conditions simultaneously within the test chamber: ambient cavity air, PVDF/TiO_2_ fabric, PEO/Clay‐UP fabric, and Mc‐sRCF, with continuous reference to ambient. Throughout the 24 h diurnal cycle, Mc‐sRCF consistently maintains the lowest temperature profile among all tested samples (Figure 6c,d). Figure 6e presents the corresponding environmental parameters recorded during the testing period, including relative humidity and solar irradiance.

During the nighttime period from 23:00 on August 5 to 06:00 on August 6, 2025 (with zero solar irradiance), Mc‐sRCF demonstrates superior cooling performance due to its high infrared emissivity, maintaining temperatures consistently below both the cavity air and ambient environment. However, the cooling effect is partially attenuated by high nocturnal humidity (peaking at 91% RH), resulting in an average temperature drop of only 5.2 °C below ambient for Mc‐sRCF. As solar irradiance increases during daytime hours, all fabric temperatures rise accordingly. The temperature differential between Mc‐sRCF and ambient air gradually decreases due to a small amount of solar absorption. From 08:00 to 16:00 on August 6, Mc‐sRCF maintains an average temperature drop of 3.9 °C relative to ambient, and reaches a maximum temperature drop of 10.0 °C (peak solar intensity: 858 W·m^−^^2^). Remarkably, owing to its exceptional solar reflectivity and mid‐infrared emissivity, Mc‐sRCF outperforms other fabrics with temperature drops of 9.4 °C (relative to cavity air), 1.1 °C (relative to PVDF/TiO_2_ fabric), and 3.7 °C (relative to PEO/Clay‐UP fabric).

The photoluminescence properties of Mc‐sRCF provide additional cooling performance enhancement by increasing ESR, thereby reducing absorbed radiation. This contributes to an outstanding average cooling power of 83.78 W·m^−^^2^, with photoluminescence accounting for 2.88 W·m^−^^2^ of this total (Figure 6f). Therefore, our Mc‐sRCF demonstrates outstanding outdoor radiative cooling performance. Table S2 summarizes the cooling performance of advanced photoluminescent radiative coolers and the contribution of photoluminescence to radiative cooling [42, 43, 44, 45, 46, 47, 48, 49, 50, 51]. These findings provide valuable insights for research on photoluminescence‐enhanced radiative cooling. Notably, our photoluminescent Mc‐sRCF boasts a competitive performance in radiative cooling, representing a critical advantage.

## 3 Conclusion

We demonstrate an environmentally stable radiative cooling fabric with exceptional durability. This fabric (Mc‐sRCF) is composed of hierarchically structured core–shell fibers incorporating multiscale TiO_2_, which synergistically optimizes solar spectral management through photoluminescent spectral shifting, multi‐interface reflection, Rayleigh scattering, and Mie scattering. This integrated approach achieves exceptional AESR reaching to 93.45%. Simultaneously, multiple infrared‐active groups (C─F, C─O─C, C─OH) combined with TiO_2_’s phonon resonance effect confer ultrahigh mid‐infrared emissivity of 95.34%. Crucially, the PVDF/TiO_2_ composite shell, featuring stable C ‐ F bonds and UV‐absorbing TiO_2_, endows the fabric with comprehensive environmental stability, including aging resistance, mechanical robustness, hydrophobicity, anti‐fouling, and flame retardancy. The Mc‐sRCF maintains uncompromised structural integrity after 12 h of accelerated UV exposure under high‐pressure mercury lamps (500 W). Field tests under Beijing summer conditions demonstrate that Mc‐sRCF achieves a daytime maximum temperature drop of 10.0°C (peak solar intensity: 858 W·m^−^^2^), with a cooling power of 83.78 W·m^−^^2^. Remarkably, the fabric shows no degradation after 60 days of prolonged outdoor exposure. The exceptional radiative cooling and environmental stability of our fabric represent a significant step forward and open up new possibilities for designing future radiative cooling technologies.

## Conflicts of Interest

The authors declare no conflict of interest.

## Supporting information




**Supporting File 1**: advs75252‐sup‐0001‐SuppMat.docx.

**Supporting File 2**: advs75252‐sup‐0002‐MovieS1.mp4.

**Supporting File 3**: advs75252‐sup‐0003‐MovieS2.mp4.

**Supporting File 4**: advs75252‐sup‐0004‐MovieS3.mp4.

**Supporting File 5**: advs75252‐sup‐0005‐MovieS4.mp4.

## Data Availability

The data that support the findings of this study are available in the supplementary material of this article.
