# Supplementary material for: Highly Efficient and Environmentally Stable Radiative Cooling Fabric: Integrating Photoluminescence and Hierarchical Core–Shell Fibers
Source: Adv Sci (Weinh). 2026 Apr 13;13(39):e75252. doi: 10.1002/advs.75252 (PMC13334863; doi:10.1002/advs.75252)
Supplement: Supplementary file 1 — Supporting File 1: advs75252‐sup‐0001‐SuppMat.docx. [file ADVS-13-e75252-s003.docx]

Supporting Information

**Highly Efficient and Environmentally Stable Radiative Cooling Fabric: Integrating Photoluminescence and Hierarchical Core-Shell Fibers**

Hongtao Liu^1,2^, Hui Li^4^, Yining Wang^1^, Wei Sun^1^, Zhuan Chen^1^, Yongping Hou^1*^, Yongmei Zheng^1*^, Xuejian Chen^3*^，Bin Yu^2*^

1 Key Laboratory of Bioinspired Smart Interfacial Science and Technology of Ministry of Education, School of Chemistry, Beihang University (BUAA), Beijing 100191, P. R. China

2 School of Transportation Science and Engineering, Beihang University (BUAA), Beijing 100191, P. R. China

3 State Key Laboratory of Advanced Waterproof Materials, Beijing Oriental Yuhong Waterproof Technology Co., Ltd, Beijing 101111, P.R. China

4 Key Laboratory of Road and Traffic Engineering of the Ministry of Education, College of Transportation, Tongji University, 4800 Cao’an Rd, Shanghai 201804, P. R. China

E-mail: houyongping09@buaa.edu.cn, zhengym@buaa.edu.cn, chenxj@yuhong.com.cn or yubinyb@buaa.edu.cn

**Contents：**

1. **Supplementary Methods**
2. **Supplementary Figure Legends: Figure S1-S17**
3. **Supplementary Notes**
4. **Supplementary Tables**
5. **Supplementary Movies: Movie S1- S4**
6. **References in Supporting Information**

**1. Supplementary Methods**

**1.1 Materials**

Polyethylene oxide (PEO, average Mv ~ 600000), Clay (Bentonite, size ≤ 25μm), Urea Phosphate (UP, 98%), Titanium dioxide (TiO_2_, 60 nm), Acetonitrile (for HPLC, ≥ 99.9%) were purchased from Shanghai Macklin BioChemical Technology Co., Ltd. Titanium oxide (TiO_2_, 500 nm) was purchased from Shanghai Dibo Biological Technology Co., Ltd. Polyvinylidene fluoride (PVDF, average Mv ~ 600000) was purchased from Shanghai yuanye Bio-Technology Co., Ltd. N-N-Dimethylformamide (DMF), Acetone (AR) and Ethanol (AR) were purchased from Modern Oriental (Beijing) Technology Development Co., Ltd. Deionized water (DI) was utilized in the experimental procedures.

**1.2** **Preparation of the PEO/Clay-UP precursor solution**

The PEO/Clay-UP precursor solution was prepared according to our previous work^[1]^. The PEO/Clay-UP precursor solution was prepared via solution mixing. Initially, Clay (5 wt.% relative to PEO) was dispersed in 20 mL of mixed solvent (deionized water: acetonitrile = 3:17). Subsequently, UP (five times the mass of Clay) was introduced, and the mixture was stirred at room temperature for 1 h. After homogenization, PEO polymer (6 wt.% relative to mixed solvent) was added and completely dissolved, followed by continuous stirring for 12 h. This yielded a viscous light-gray precursor solution, designated as PEO/Clay-UP.

**1.3** **Preparation of the PVDF/TiO_2_ precursor solution**

The PVDF/TiO₂ precursor solution was prepared using an analogous solution-mixing protocol. Initially, multiscale TiO₂ nanoparticles (4 wt.% relative to PVDF polymer, 60 nm:500 nm = 2:1) were dispersed in 20 mL of mixed solvent (DMF: acetone= 3:7) with continuous stirring at room temperature for 1 h. Following homogenization, PVDF powder (12 wt.% relative to total solvent) was introduced and dissolved under intensive mechanical stirring. The mixture subsequently underwent ultrasonication for 30 min followed by 12 h stirring at ambient temperature, yielding a homogeneous white PVDF/TiO₂ precursor solution.

**1.4 Preparation of the Mc-sRCF**

The Mc-sRCF was fabricated via coaxial electrospinning. Precursor solutions of PEO/Clay-UP and PVDF/TiO₂ were loaded into separate 20 mL syringes as core and shell fluids, respectively. A custom coaxial spinneret with 21-gauge inner and 16-gauge outer needles was employed. The PEO/Clay-UP core solution and PVDF/TiO₂ shell solution were co-electrospun at a positive voltage of 16 kV (needle) and a negative voltage of 1 kV (collector) with a core/shell flow rate ratio of 3:7. Fibers were collected on aluminum foil wrapped around a rotating drum (500 rpm) positioned 15 cm from the spinneret. Critically, the spinning environment temperature was adjusted to 25^o^C and the humidity was 52 ± 2% RH to optimize fiber morphology. The resultant fabric was subsequently dried at 40^o^C for 10 h in a convection oven to remove residual solvents.

**1.5 Morphology, wettability, and elemental characterization**

A scanning electron microscope (SEM, Tescan XEIA) was used to characterize the apparent morphology and structure of the fabrics. A transmission electron microscopy (TEM, JEM F200) was examined the internal structure of the fibers and obtain elemental maps by performing energy dispersive spectroscopy (EDS). The wettability of various fabrics was characterized by measuring static contact angles using an optical contact angle goniometer system (OCA 40 Micro, DataPhysics Instruments GmbH) with 3 μL deionized water droplets at ambient conditions (25^o^C, 50% RH). Fourier transform infrared (FTIR) spectra ranging from 4000 to 600 cm^-1^ were obtained using an FTIR spectrometer (Frontier, PE) equipped with an attenuated total refraction (ATR) module. X-ray photoelectron spectroscopy (XPS) measurement was performed using a Thermo Scientific K-Alpha instrument with monochromated Al Kα radiation (hν = 1486.6 eV) under ultrahigh vacuum (~5 × 10⁻⁹ mbar) to quantify elemental distributions. The textural characteristics of the fabric, including pore diameter distribution and specific surface area, were determined by nitrogen adsorption/desorption measurements using an automated surface area and porosity analyzer (BSD-660, Beishide Instrument) employing the Brunauer-Emmett-Teller (BET) method.

**1.6 Optical characterization**

The solar reflectance (0.3-2.5 µm) of the fabrics was measured using an ultraviolet-vis/near-IR (UV-VIS/NIR) spectrophotometer (UH4100, Hitachi) with an integrating sphere. The reflectivity in the solar spectrum between wavelengths $\text{λ}_{\text{1}}$to $\text{λ}_{\text{2}}$ can be defined as Eq. (S1):

$\text{R}_{\text{Solar}}\text{=}\frac{\int_{\text{λ}_{\text{1}}}^{\text{λ}_{\text{2}}} \text{I}_{\text{AM1.5}}\left( \text{λ} \right)\text{⋅}\text{R}\left( \text{λ} \right)\text{dλ}}{\int_{\text{λ}_{\text{1}}}^{\text{λ}_{\text{2}}} \text{I}_{\text{AM1.5}}\text{dλ}}$ (S1)

where, $\text{I}_{\text{AM1.5}}\text{ }\text{R}\text{(λ)}$ is the standard specular solar irradiance with an overall intensity of approximately 1,000 W/m^2^, $\text{R}\text{(λ)}$ is the reflectance of the fabric at $\text{λ}$ wavelength.

A Fourier transform infrared spectrometer (Nicolet iS50, Thermo Field) with a gold-coated integrating sphere was used to measure the absorption/emissivity of the cooling fabrics in the wavelength range of 4-16 µm. Similarly, the mid-infrared (MIR) emission can be defined as Eq. (S2):

$\text{ε}_{\text{MIR}}\text{=}\frac{\int_{\text{8}}^{\text{13}} \text{ }\text{I}_{\text{bb}}\text{(λ,T)}\text{⋅}\text{ε(λ,T)dλ}}{\int_{\text{8}}^{\text{13}} \text{ }\text{I}_{\text{bb}}\text{(λ,T)dλ}}$ (S2)

$\text{I}_{\text{bb}}\text{(}\text{λ,T}\text{) }$is the spectral emissive power of a black body. It can be expressed as the Planck formula, that $\text{I}_{\text{bb}}\text{(}\text{λ,T}\text{)=}\frac{\text{2h}\text{c}_{\text{0}}^{\text{2}}}{\text{λ}^{\text{5}}\left[ \text{exp}\left( \text{h}\text{c}_{\text{0}}\text{∕λ}\text{κ}_{\text{b}}\text{Τ} \right)\text{-1} \right]}$. In which,$\text{ h=6.626×}\text{10}^{\text{-34}}\text{ }\text{J}\text{⋅}\text{s}$ is the universal Plank constant, $\text{κ}_{\text{b}}\text{=1.381×}\text{10}^{\text{-23}}\text{ J·}\text{K}^{\text{-1}}$ is the Boltzmann constant, and $\text{c}_{\text{0}}\text{=2.998×}\text{10}^{\text{8}}\text{ }\text{m·}\text{s}^{\text{-1}}$ is the speed of light in vacuum.

Steady-state/transient fluorescence spectrometer (EFLS1000, Edinburgh Instruments) was employed to characterize the excitation/emission spectra, luminescence lifetimes (τ), and quantum yields (Φ) of both fluorescence and phosphorescence under nitrogen atmosphere.

**1.7 Mechanical behavior test**

The thickness of the fabricated fabrics was measured using an electronic vernier calliper (150 T, MNT). Uniaxial tensile and cyclic tensile tests of the cooling fabrics were conducted using a universal testing machine (CTM 2500, XieQiang Instrument). Samples were cut to dimensions of 80 mm × 10 mm × 1 mm and were stretched at a speed of 100 mm/min. The tests were repeated five times to obtain an average value. Under a vertical pressure of 0.588 N, the fabrics were repeatedly slid horizontally against 2000-grit silicon carbide (SiC) sandpaper (280 × 230 mm) at a speed of 6 m/s for 100 cycles to evaluate its abrasion resistance.

**1.8 Thermal stability test**

Thermogravimetric analysis (TGA, Rigaku TG/DTA 8122) was performed under a nitrogen atmosphere from 30 to 800°C at a heating rate of 10°C/min to evaluate the thermal degradation behavior of the fabric. Differential scanning calorimetry (DSC, TA DISCOVERY DSC2500) was conducted from 25 to 500°C at 10°C/min under nitrogen to analyze endothermic/exothermic transitions (e.g., melting, pyrolysis) and heat capacity changes in the fabric.

**1.9 Laboratory Cooling performance test:**

The radiation cooling performance of the cooling fabrics was tested in laboratory, where a solar simulator was used to directly irradiate the surface of the cooling fabrics. The change of samples temperature over time was recorded by a thermocouple (TA612C, TASI). Thermal infrared imaging of the samples was recorded over time with a thermal infrared camera (A655sc, ATS).

**1.10 Environmental stability tests**

The fabric's durability was evaluated through long-term outdoor exposure testing under various weather conditions (including clear, cloudy, rainy, and windy days), with systematic monitoring of surface morphological changes and mass loss over time. The anti-fouling properties of the fabrics were evaluated through controlled mud solution contamination tests, with subsequent assessment of surface cleanliness using high-resolution digital imaging (Canon 90D, Canon Inc.).

**1.11 Outdoor Cooling performance test**

Examinations of the fabrics’ cooling performance were conducted in Beijing, China (40.1512°N, 116.2793°W; 46 m altitude). The radiation cooling performance of the cooling fabrics was tested using a homemade device (the test box size is 30 cm × 30 cm × 30 cm). The samples were cut into a size of 8 cm × 8 cm × 8 cm and placed in the cavity of the cooling test box. The change of samples temperature over time was recorded by a thermoCouple (TA612C, TASI), the solar power meter (TES-1333R, TES) recorded the solar radiation intensity of the samples, the temperature and humidity recorder (COS-03, JianDaRenKe) recorded the temperature and humidity, and the anemometer (AS8336, SMART SENSOR) recorded the wind speed outside the test box.

**1.12 Theoretical cooling power**

The net radiant cooling power is a calculation standard used to measure the degree of cooling of an object. The net radiative cooling power is determined as Eq. (S3):

$\text{P}_{\text{cooling}}\text{=}\text{P}_{\text{rad}} \text{-}\text{ }\text{P}_{\text{sun}} \text{-}{\text{ }\text{P}}_{\text{amb}} \text{-}{\text{ }\text{P}}_{\text{conv+cond}}$ (S3)

in which, $\text{P}_{\text{cooling}}\text{ }$is defined as net radiative cooling power, $\text{P}_{\text{rad}}$ is defined as the infrared radiation power emitted by the cooling fabrics, and $\text{P}_{\text{sun}}$ is defined as denotes the spectral power of sunlight. $\text{P}_{\text{conv+cond}}$ is the loss of cooling power due to convection and conduction.

The $\text{P}_{\text{rad}}\text{ }$can be expressed as Eq. (S4):

$\text{R}_{\text{rad}}\text{=σ}\text{Τ}^{\text{4}}\frac{\int\text{I}_{\text{bb}}\text{(λ,T)}\text{⋅}\text{ε(λ,T)dλ}}{\int\text{I}_{\text{bb}}\text{(λ,T)dλ}}$ (S4)

in which, $\text{σ}$ is the Stefan-Boltzmann constant and $\text{ε}\left( \text{λ,}\text{ }\text{T} \right)\text{ }$is the mid-infrared spectral emissivity of cooler. $\text{I}_{\text{bb}}\text{(}\text{λ,T}\text{) }$is the spectral emissive power of a black body.

The $\text{P}_{\text{sun}}\text{ }$can be expressed as Eq. (S5):

$\begin{aligned} \text{P}_{\text{sun}}\text{=}\text{P}_{\text{absorption }}\text{-}\text{ P}_{\text{emission}} \\ \text{=}\text{(1-ESR)P}_{\text{solar }} \end{aligned}$ (S5)

in which, effective solar reflectance (ESR) is derived from the photoluminescence QY (Figure Note1), and the Psolar is solar radiation intensity.

The $\text{P}_{\text{amb}}\text{ }$can be expressed as Eq. (S6):

$\text{P}_{\text{amb}}\text{=σ}\text{T}_{\text{amb}}^{\text{4}}\frac{\text{∫}{\text{I}_{\text{bb}}\left( \text{λ,T} \right)\text{⋅}\text{ε}}_{\text{amb}}\text{(λ,T)dλ}}{\text{∫}\text{I}_{\text{bb}}\text{(λ,T)}\text{⋅}\text{dλ}}$ (S6)

in which, $\text{ε}_{\text{amb}}$ is the mid-infrared spectral emissivity of ambient air. $\varepsilon_{\mathrm{amb}}$ is assumed to be 0.725.

The $\text{P}_{\text{conv+cond}}\text{ }$can be expressed as Eq. (S7):

$\text{P}_{\text{conv+cond}}\text{=h(}\text{T}_{\text{amb}}\text{-}\text{T}_{\text{c}}\text{)}$ (S7)

In which $\text{h}$ is the thermal transfer coefficient between the ambient and the cooler in the forms of conduction together with convection, that $\text{h=}\text{qA}\text{=}\left( \text{2.8+3.0}\text{μ}_{\text{α}} \right)\text{A}$. In the radiative cooling test, we assume that there is no heat transfer from surrounding objects and the environment, so h is assumed to be 0 W·m^-2^.

**2. Supplementary Figure Legends**

**
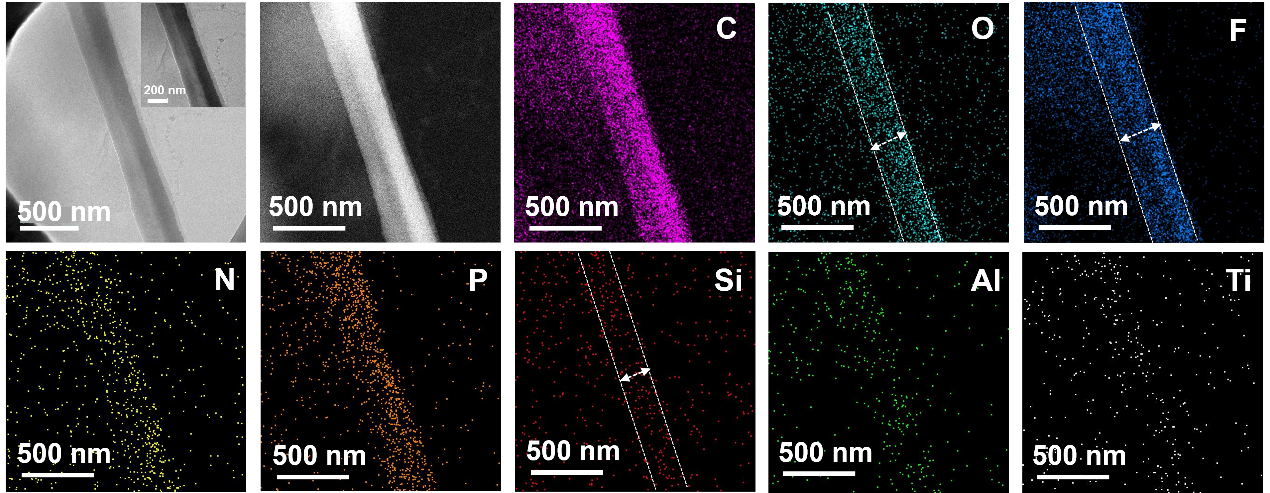
**

**Figure S1.** Transmission electron microscopy (TEM) analysis of a single Mc-sRCF fiber reveals its three-layered hierarchical core-shell structure. (The F element is attributed to the outer-layer PVDF, the O element originates from the middle-layer PEO, and the Si element is derived from the inner-layer Clay). The mass percentages of the elements C, O, F, N, P, Si, Al, and Ti are 87.84%, 3.03%, 7.86%, 0.45%, 0.60%, 0.16%, 0.02%, and 0.05%, respectively.

**
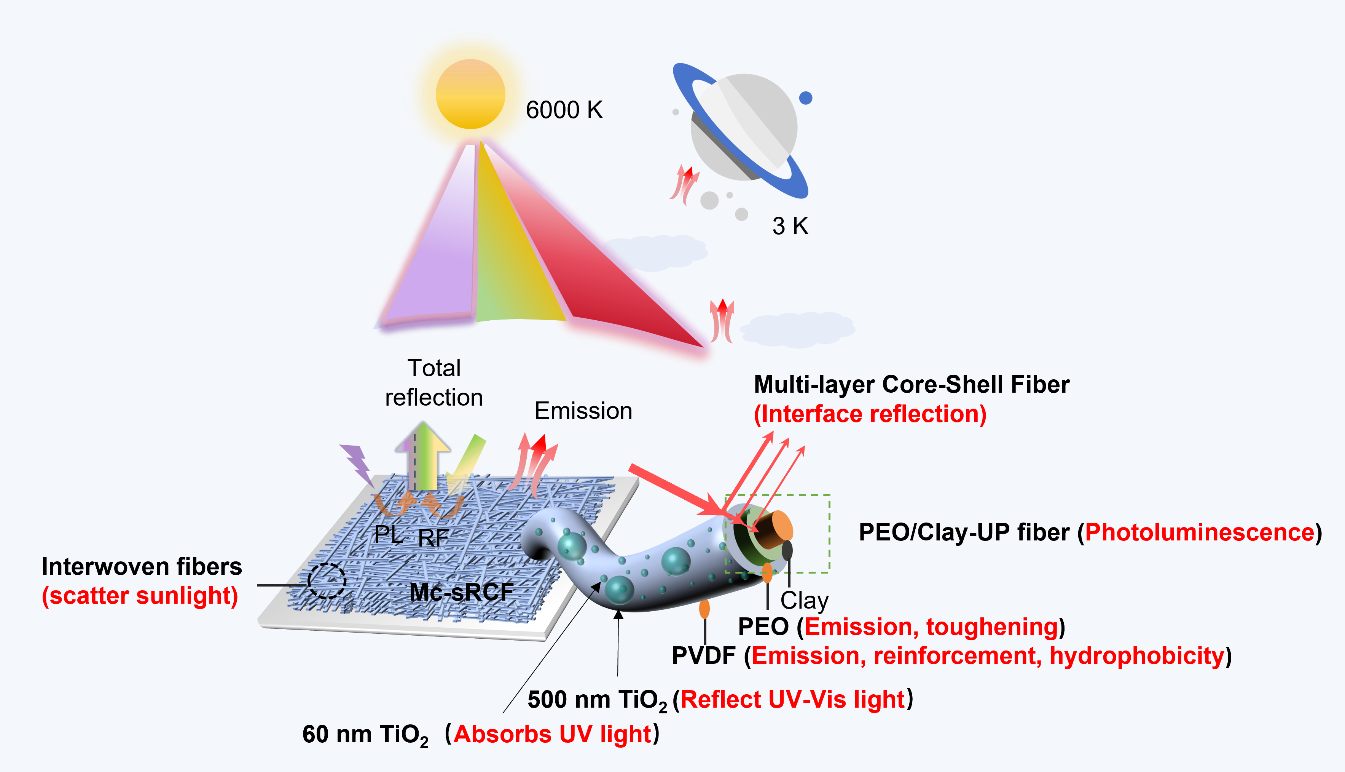
**

**Figure S2.** Functional illustration of the individual components within the Mc-sRCF fabric.


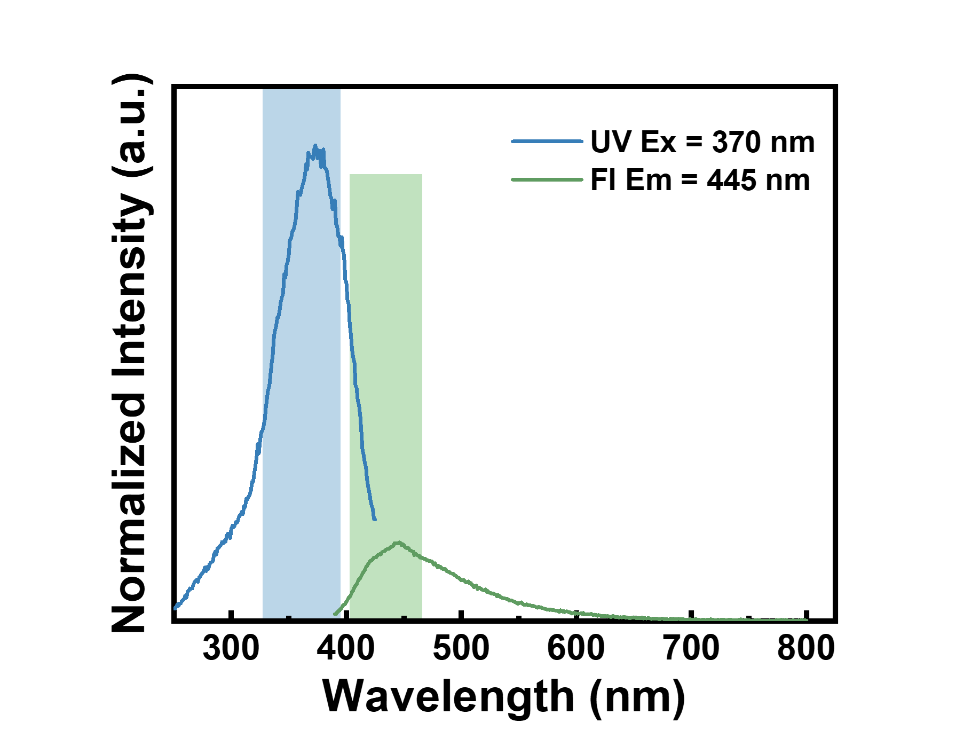


**Figure S3.** Fluorescence (445 nm) emission spectra of PEO under 370 nm UV excitation. The low emission intensity indicates the absence of significant photoluminescence behavior of PEO.

**
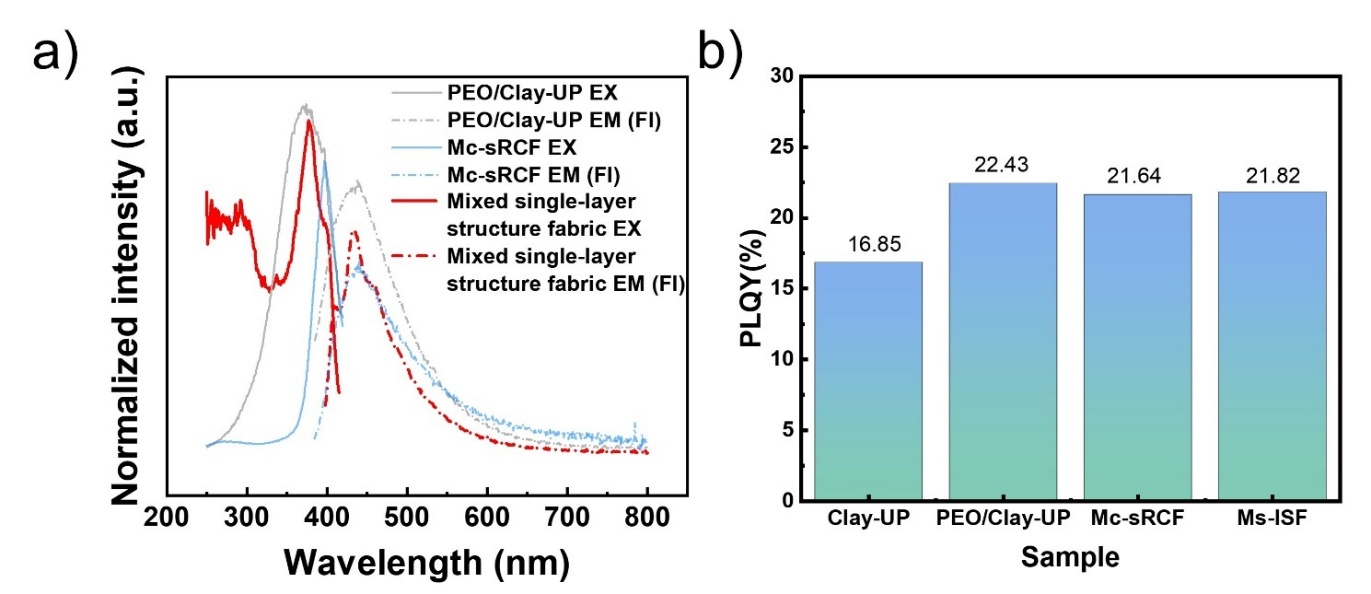
**

**Figure S4.** Photoluminescence properties of the fabrics: (a) Excitation-emission spectra and (b) absolute quantum yield. The mixed single-layer structure fabric (Ms-lsf) exhibited an emission peak at 435 nm higher than that of the Mc-sRCF fabric but lower than that of the PEO/Clay-UP fabric. Similarly, the absolute photoluminescence quantum yield (PLQY) of Ms-lsf fell between those of the PEO/Clay-UP fabric and the Mc-sRCF fabric. These observations result from the higher fluorescence emissivity of the exposed PEO/Clay-UP fibers present in the mixed structure, in contrast to their encapsulation within the multilayer core-shell architecture.


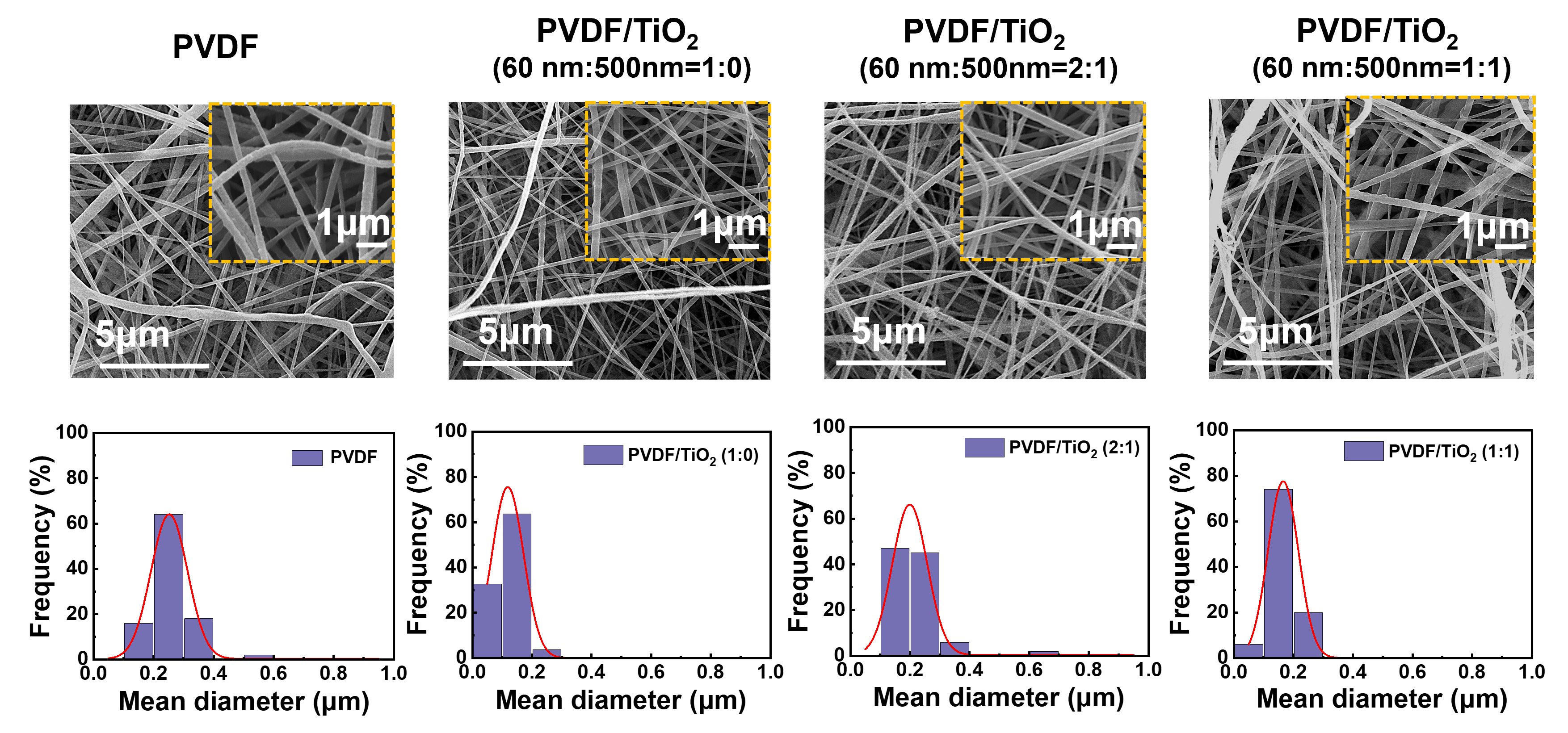


**Figure S5.** SEM morphology and fiber diameter distribution of PVDF/TiO₂ fabrics incorporated with TiO₂ particles of different size ratios (60 nm: 500 nm). The addition of TiO₂ nanoparticles with different size ratios does not significantly affect the fiber diameter, and all fibers had an average diameter below 500 nm.


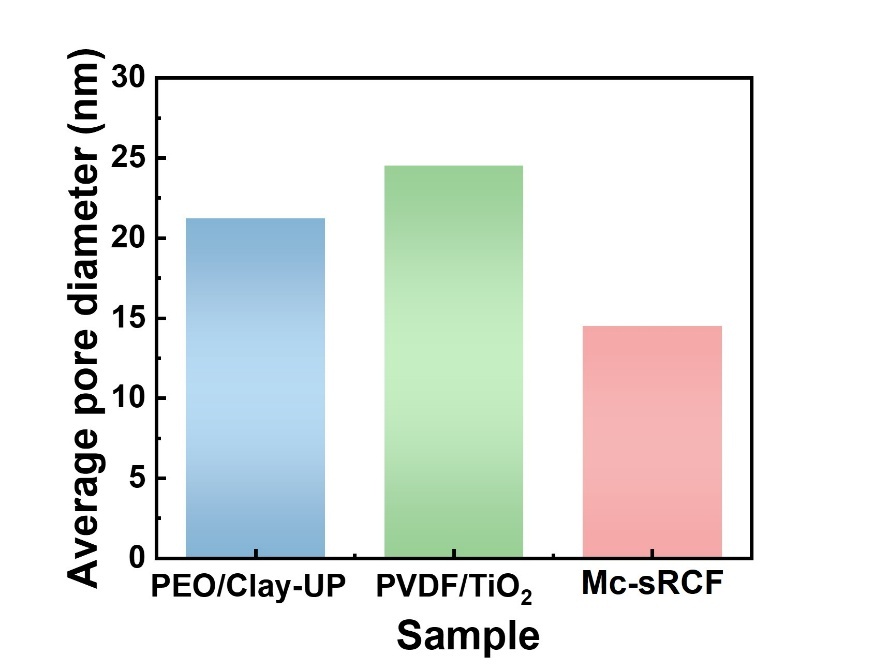


**Figure S6.** Average pore sizes of PEO/Clay-UP fabric, PVDF/TiO₂ fabric, and Mc-sRCF. The results indicate that Mc-sRCF possesses the smallest pore size.

**
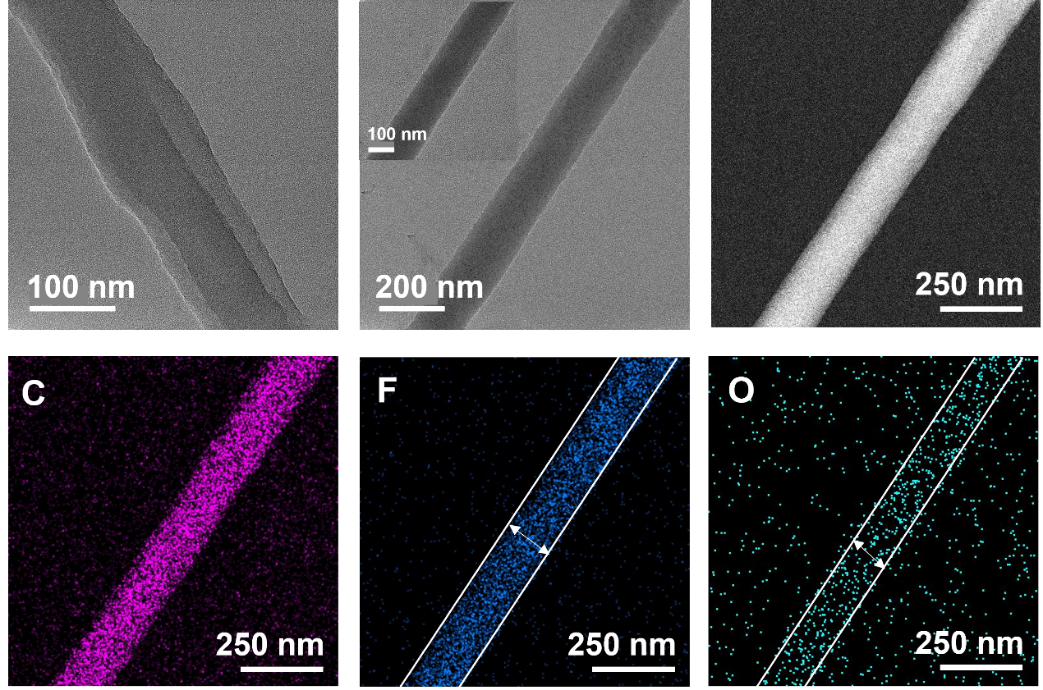
**

**Figure S7.** Transmission electron microscopy (TEM) analysis of a single core-shell fiber (PEO core/PVDF shell) reveals its two-layered hierarchical core-shell structure (The F element is attributed to the shell-layer PVDF, the O element originates from the core-layer PEO).


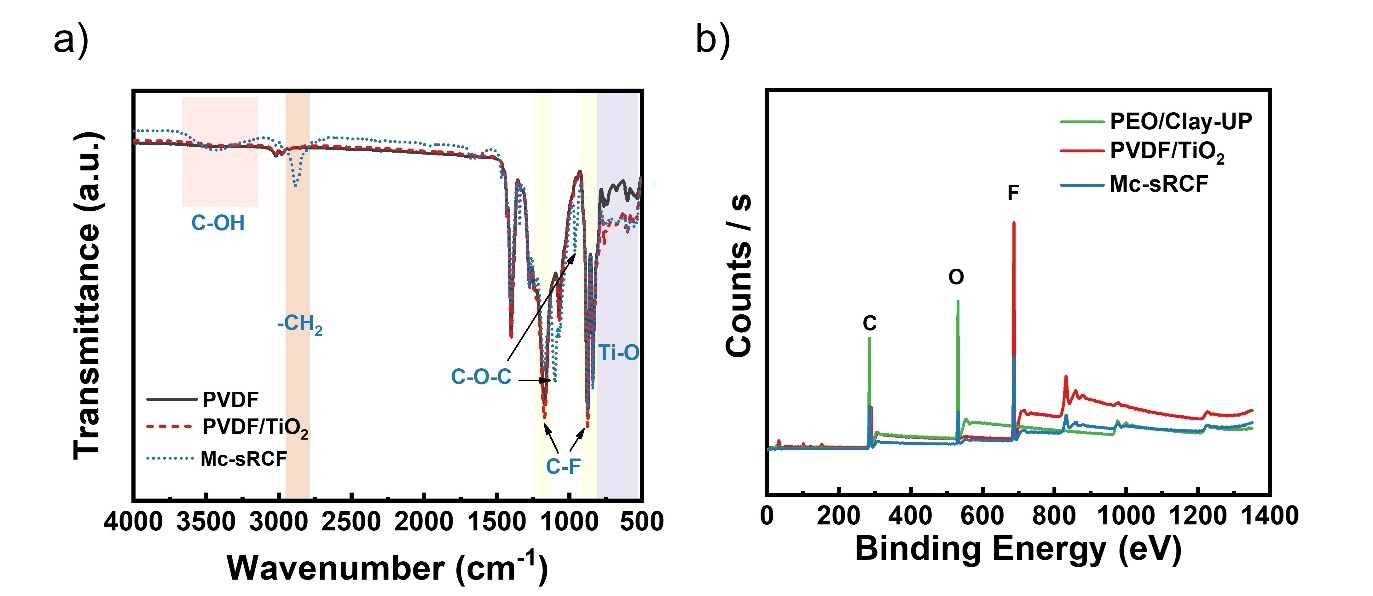


**Figure S8.** Compositional and structural analysis of the fabrics: **a)** Fourier-transform infrared (FTIR) spectroscopy and **b)** X-ray photoelectron spectroscopy (XPS) analysis. The Mc-sRCF exhibits all characteristic bonds and atoms from both PEO and PVDF, which accounts for its high infrared emissivity.

**
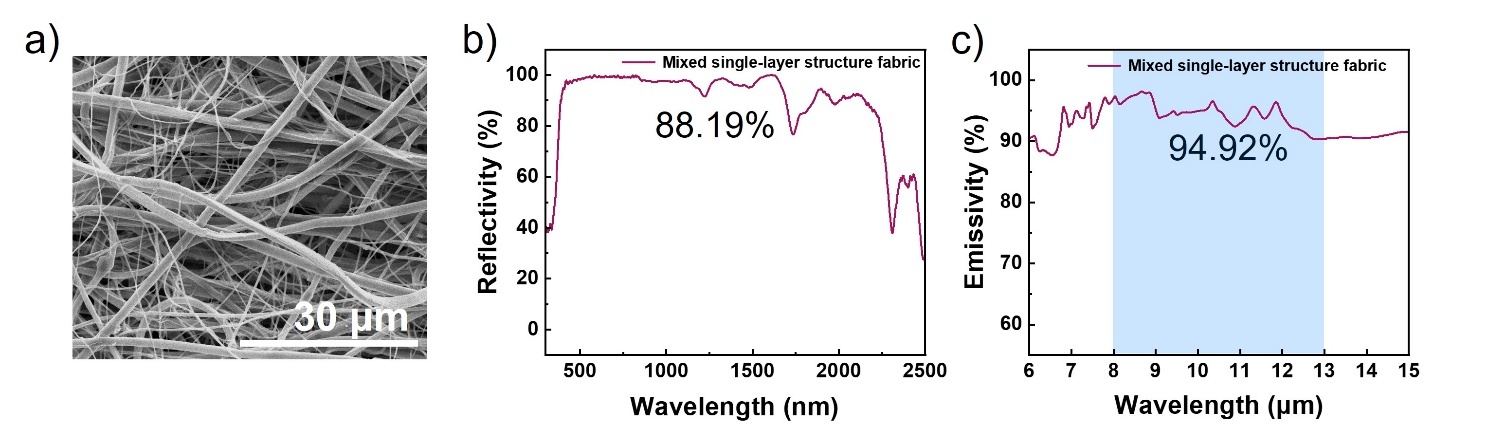
**

**Figure S9**. Micromorphology and spectral performance of the mixed single-layer structure fabric: a) SEM image, b) solar reflectance, and c) infrared emissivity. The mixed single-layer structure fabric achieves a solar reflectance of 88.19% and an infrared emissivity of 94.92%, both substantially lower than those of our Mc-sRCF fabric, underscoring the superior spectral regulation capability of multi-layered core-shell structure in Mc-sRCF.


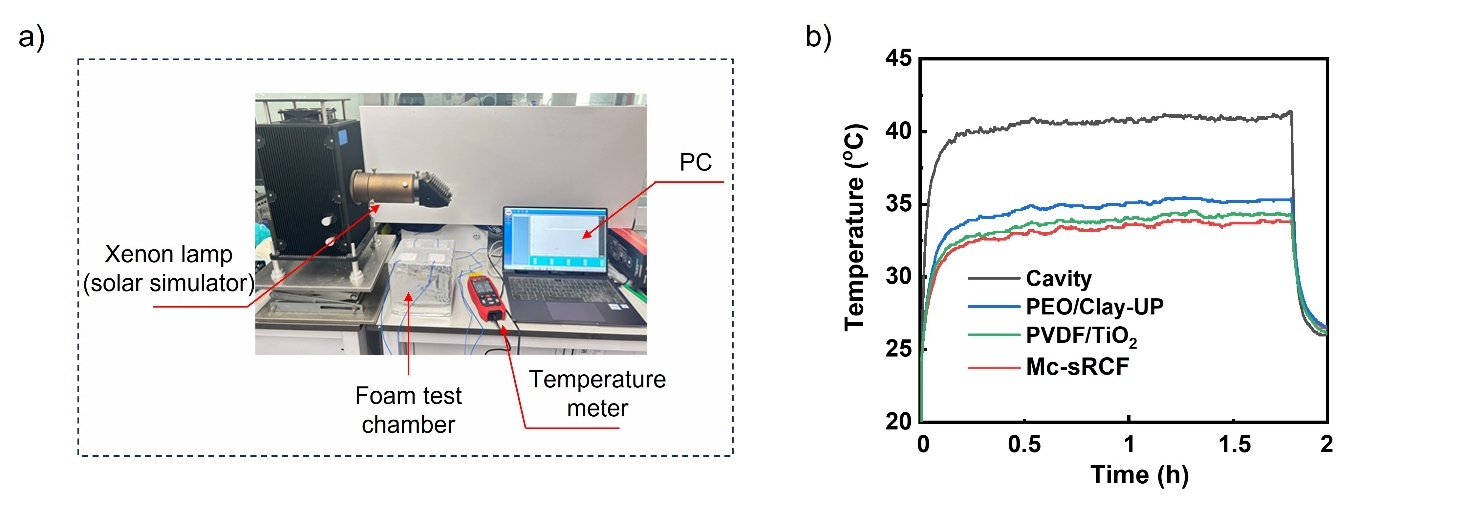


**Figure S10.** Experimental evaluation of radiative cooling performance: **a)** Schematic of the test apparatus and **b)** Temporal temperature profile under a solar irradiance of 1000 W/m². The Mc-sRCF achieves an average temperature reduction that is 9.2^o^C, 2.8^o^C, and 1.6^o^C lower than that of the cavity, PEO/Clay-UP fabric, and PVDF/TiO₂ fabric, respectively. It demonstrates superior cooling performance of Mc-sRCF among comparable fabrics.


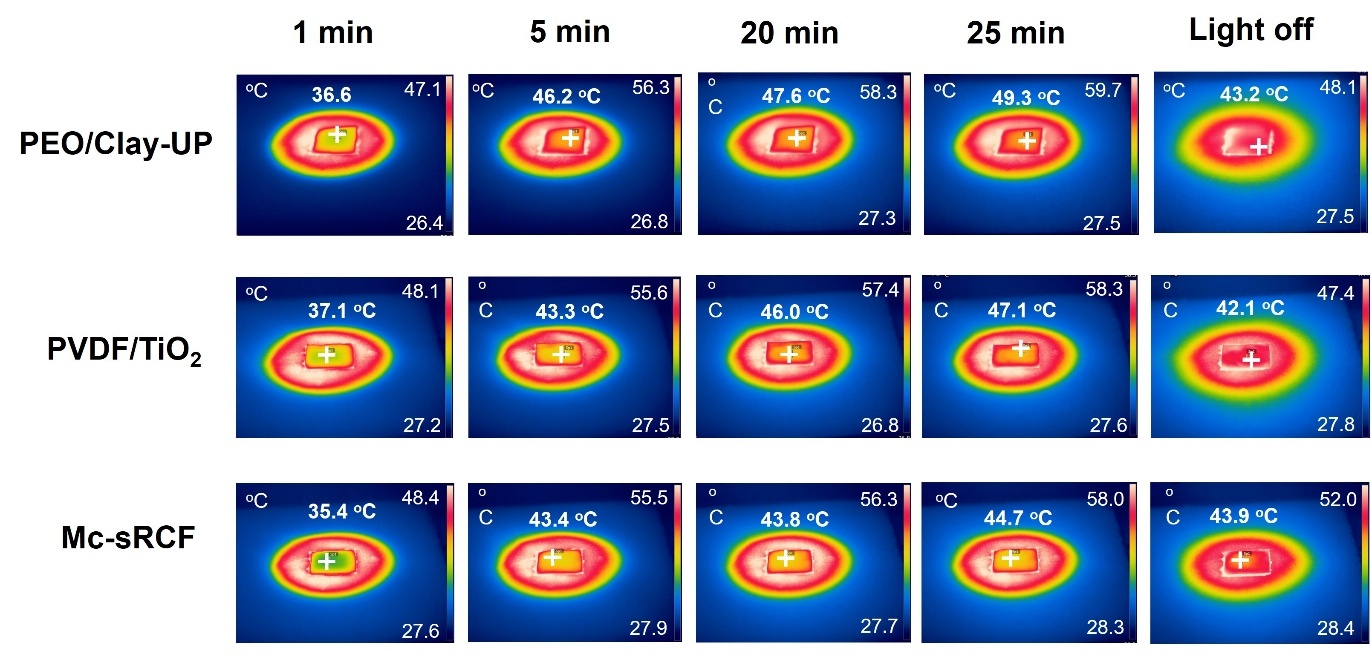


**Figure S11.** Time-resolved infrared thermography of PEO/Clay-UP fabric, PVDF/TiO_2_ fabric and Mc-sRCF under a solar irradiance of 1275 W/m², showing temperature evolution dynamics. The Mc-sRCF (44.7^o^C) achieves an average temperature reduction that is 4.6^o^C and 2.4^o^C lower than that of the PEO/Clay-UP fabric (49.3 °C) and the PVDF/TiO₂ fabric (47.1^o^C), respectively. It demonstrates the excellent radiative cooling performance of Mc-sRCF even under high solar intensity.

**Figure S12.** UV absorption spectra of PVDF fabrics incorporated with TiO₂ nanoparticles at varying size ratios (60 nm:500 nm).


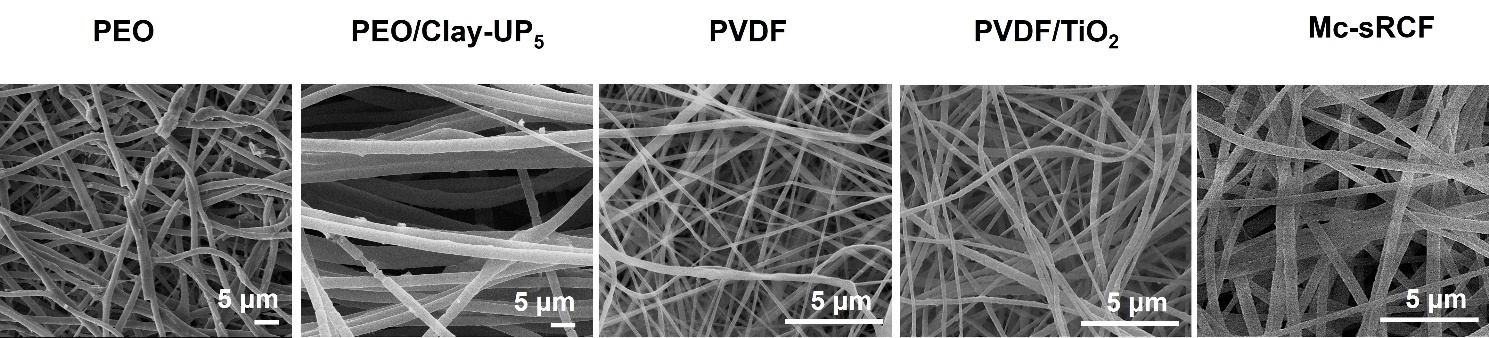


**Figure S13.** SEM morphologies of PEO fabric, PEO/Clay-UP fabric, PVDF fabric, PVDF/TiO_2_ fabric and Mc-sRCF, after 6 h of UV high-pressure mercury lamp (500W) irradiation. The PEO fabric shows fiber breakage, whereas the other fabrics remain intact.

**
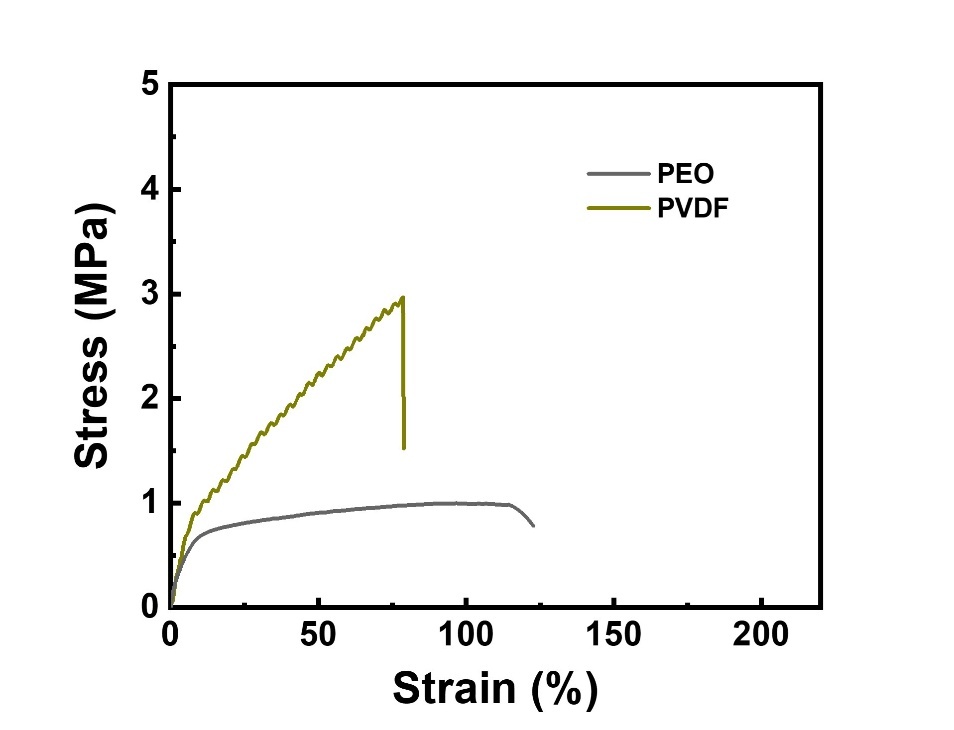
**

**Figure S14.** Stress-strain curves of PEO and PVDF fabrics.

**
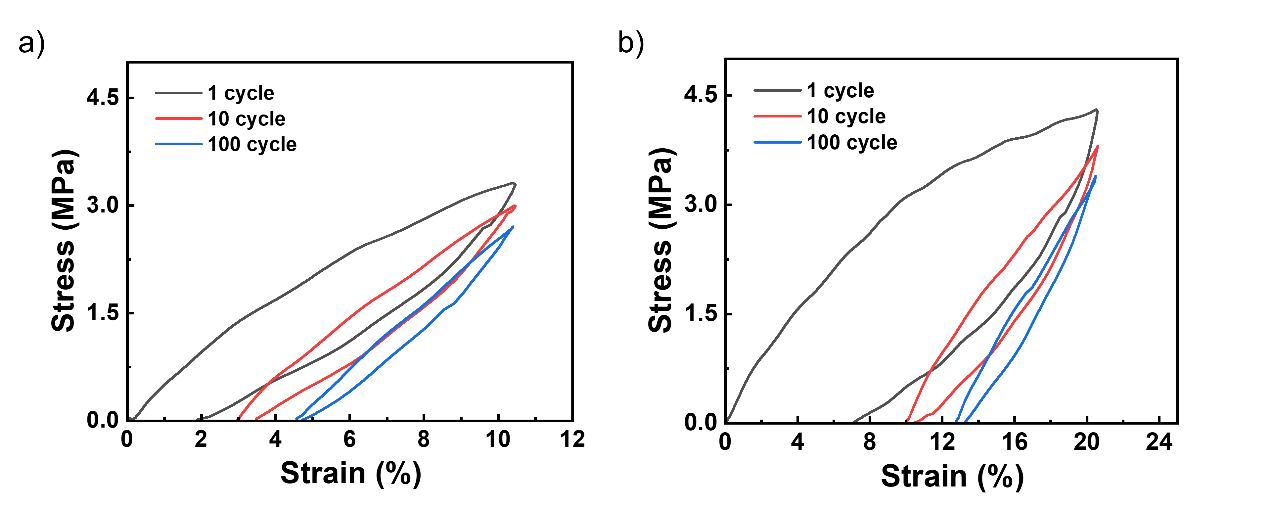
**

**Figure S15.** Cyclic tensile stress-strain curves of Mc-sRCF over 100 loading-unloading cycles at **(a)** 10% and **(b)** 20% strain after 12 hours of UV irradiation (500 W). It demonstrates outstanding mechanical stability of Mc-sRCF.


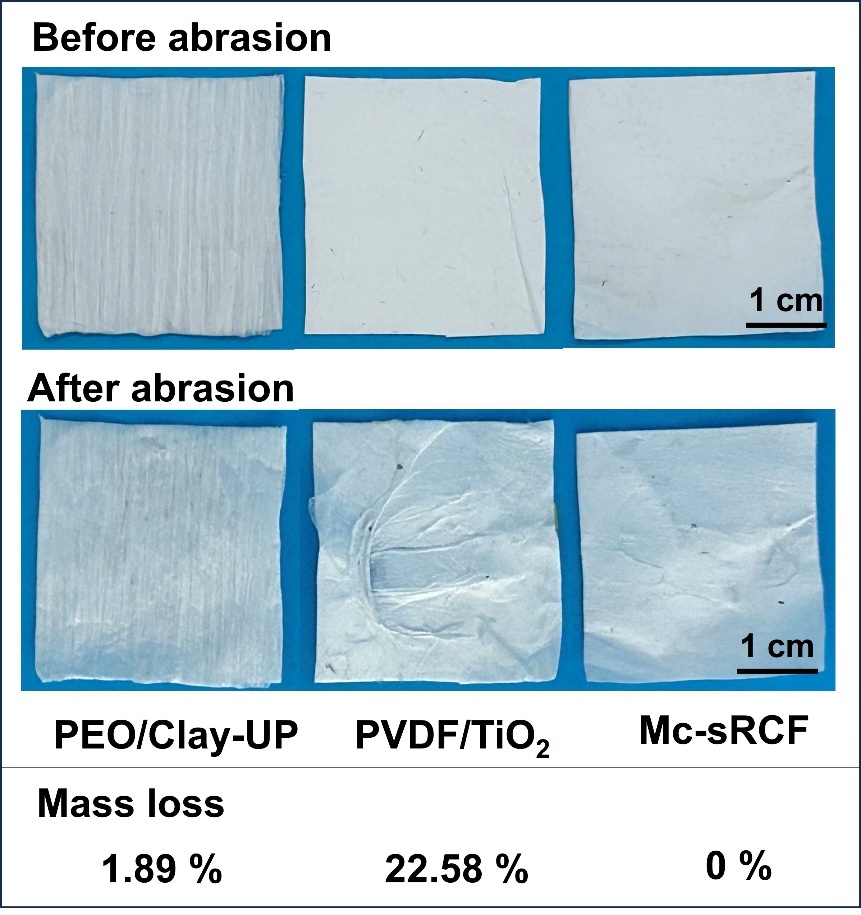


**Figure S16.** Photographic comparison of PEO/Clay-UP fabric, PVDF/TiO_2_ fabric and Mc-sRCF before and after abrasion testing, along with the corresponding mass loss data. The Mc-sRCF exhibits the least abrasion and the lowest mass loss, demonstrating its superior abrasion resistance.


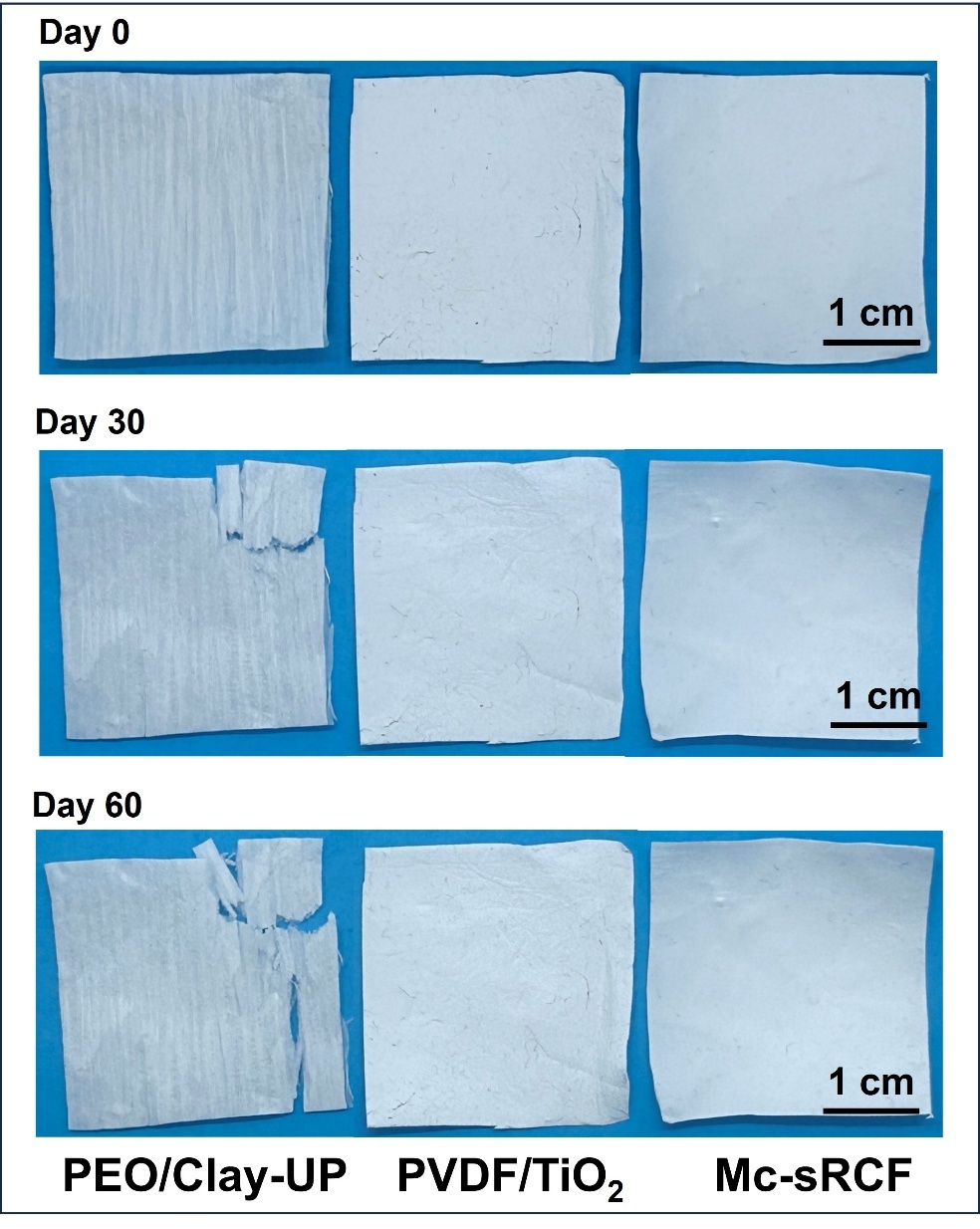


**Figure S17**. Evaluation of fabrics degradation under outdoor exposure conditions. The PEO fabric begins to degrade by day 30 and shows severe degradation by day 60, whereas the PVDF/TiO₂ fabric and Mc-sRCF remain morphologically intact with no obvious signs of degradation.


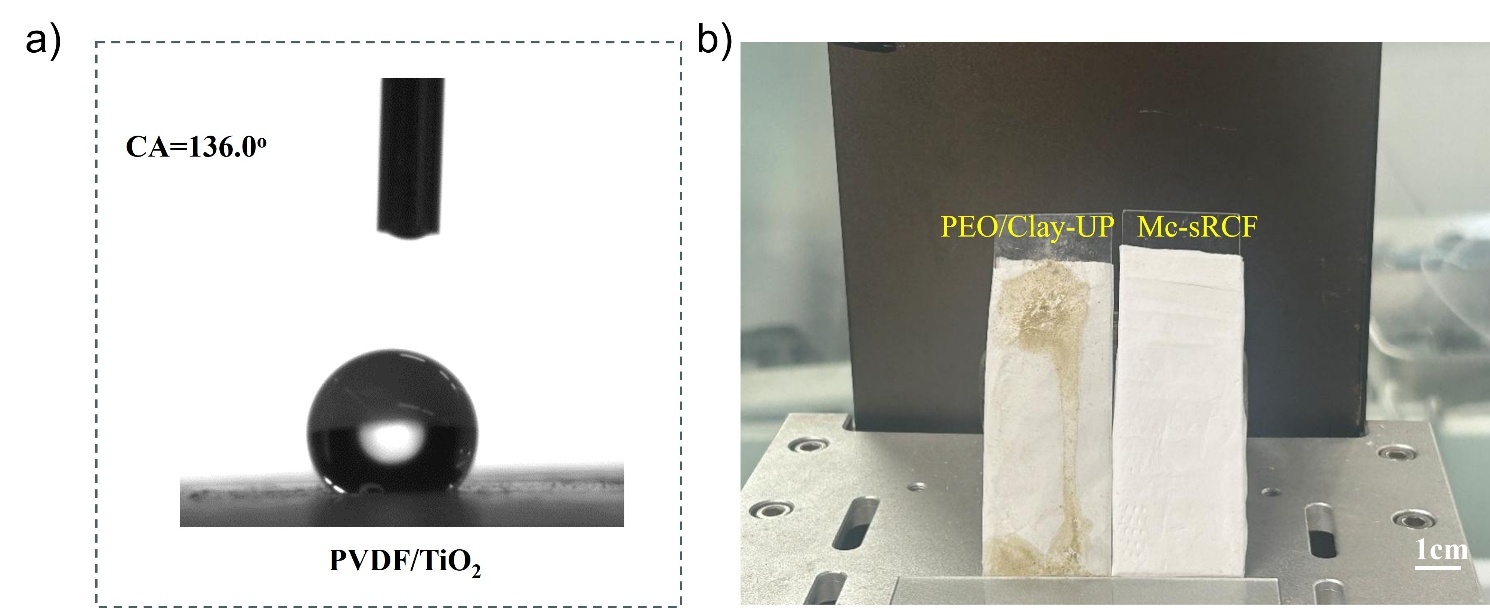


**Figure S18.** a) Contact angle image of PVDF/TiO_2_ fabric. b) Digital image of PEO/Clay-UP fabric (left) and Mc-sRCF (right) after muddy water erosion. Muddy wastewater droplets slide down the fabric surfaces. The PEO/Clay-UP fabric is covered with contamination stains, whereas the Mc-sRCF surface remains clean, confirming the excellent water-repellent and anti-fouling properties of Mc-sRCF.


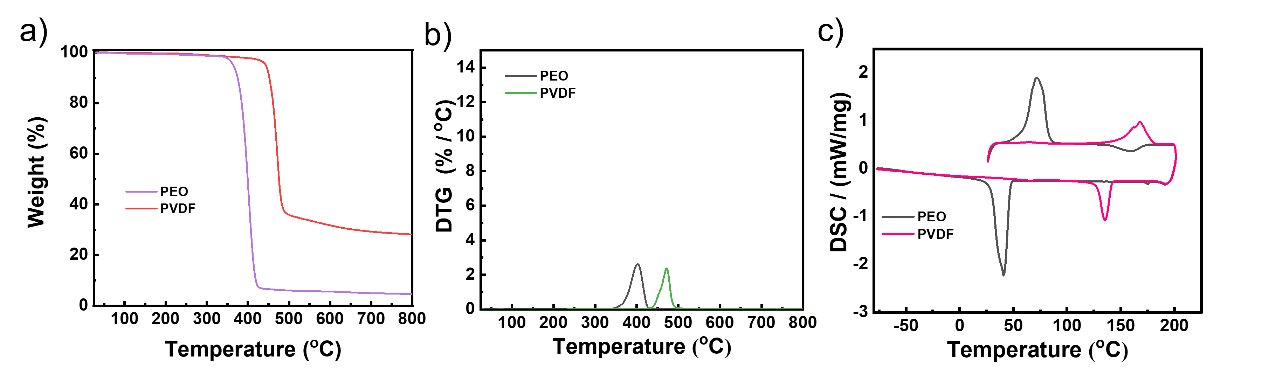


**Figure S19.** Thermal stability analysis of PEO and PVDF fabrics, including TGA, DTG, and DSC thermograms.


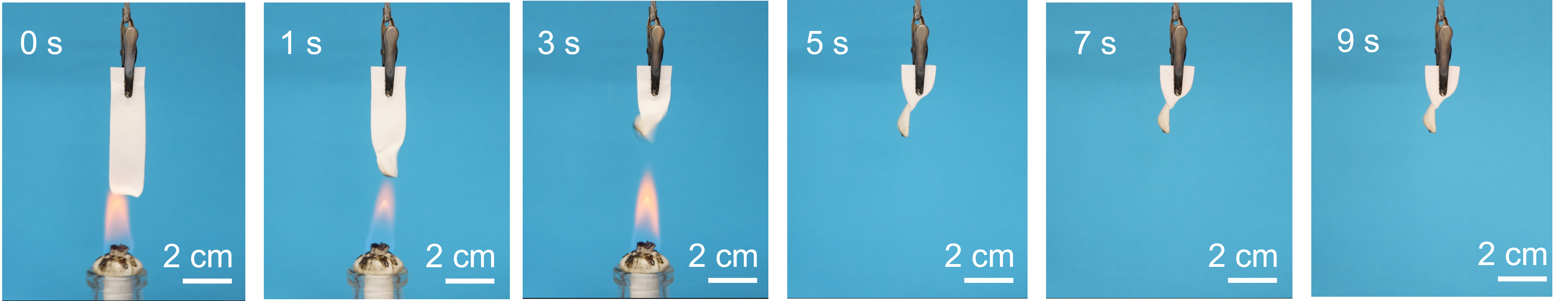


**Figure S20.** Time-dependent combustion behavior of PVDF/TiO_2_ fabric, demonstrating effective flame retardancy.


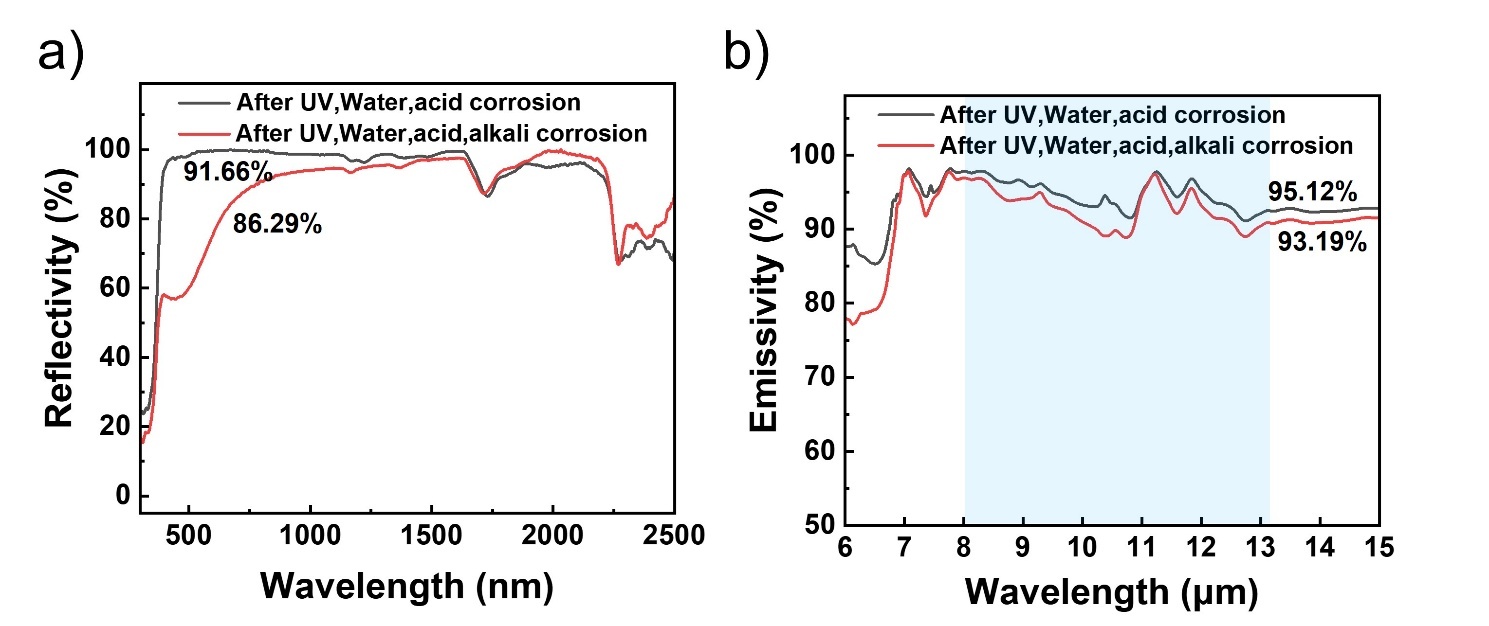


**Figure S21.** The optical performance of the Mc-sRCF fabric after sequential exposure to UV radiation, water immersion, acid and alkali erosion for 12 hours each. The results show that after exposure to UV, water, and acid, the fabric maintained a high solar reflectance of 91.66% and an infrared emittance of 95.12%, demonstrating excellent environmental durability. However, following further immersion in a sodium hydroxide solution with a pH of 13, the solar reflectance and infrared emittance experienced slight decreases to 86.29% and 93.19%, respectively. This minor degradation is primarily attributed to the partial disruption of the PVDF molecular chain structure under strong alkaline conditions, which marginally compromises the material's optical regulation capability. Nevertheless, the fabric still retains a relatively high spectral selectivity, further confirming the overall environmental stability of the Mc-sRCF fabric under complex conditions.

**3. Supplementary Notes**

**3.1. Concepts, theoretical calculations, and testing methods of effective solar reflectance (ESR)**

Fluorescent radiative coolers can maintain sub-ambient temperatures even under direct solar irradiation. This cooling mechanism relies on three critical processes: (1) high mid-infrared (NIR) emissivity, (2) broadband solar reflectance, and (3) fluorescence-mediated re-emission of absorbed visible light. The cooling performance is quantified by the effective solar reflectance (ESR, related to the processes (2) and (3)) defined as the total fraction of incident solar energy rejected through both reflection and fluorescence emission. However, conventional optical instruments, such as solar spectrometers and reflectometers, cannot accurately measure ESR, as they are designed only for standard solar reflectance (SR) measurements. ESR measurement techniques can be classified into two primary categories: radiometric methods (based on direct radiation measurement) and calorimetric methods (based on thermal response analysis). Herein, we employ calorimetric methods to determine both the ESR and effective solar absorptance (ESA, ESA = 1 – ESR, for non-transparent materials where transmission losses can be neglected).

**3.1.1 Calorimetric calculation of ESA and ESR**

Thermal equilibrium of the irradiated surface was modeled using classical linearized radiation theory (300K blackbody background), yielding steady-state temperature (T) through detailed energy balance considerations:

$$\begin{aligned} \text{α}_{\text{e}}\text{I=}\text{h}_{\text{c}}\left( \text{T-}\text{T}_{\text{a}} \right)\text{+}\text{h}_{\text{r}}\left( \text{T-}\text{T}_{\text{r}} \right)\#\left( \text{S}\text{8} \right) \end{aligned}$$

where $\alpha_{e}$ represents the effective solar absorptivity (ESA), $I$ denote the solar irradiance (W/m²), $T_{a}$ is the ambient air temperature, and $T_{r}$ corresponds to the radiative exchange temperature (approximately equivalent to sky temperature for outdoor conditions). The coefficients $h_{c}$ and $h_{r}$ represent the convective and radiative heat transfer coefficients, respectively. Since our testing apparatus was meticulously designed, convective heat transfer can be safely neglected (i.e., $h_{c}$= 0).

So,

$$\begin{aligned} \text{α}_{\text{e}}\text{I=}\text{h}_{\text{r}}\left( \text{T-}\text{T}_{\text{r}} \right)\#\left( \text{S}\text{9} \right) \end{aligned}$$

A direct linear proportionality between $\text{α}_{\text{e}}$ and $\text{T-}\text{T}_{\text{r}}$ is established in Equation S2, permitting quantitative assessment of $\text{α}_{\text{e}}$ through systematic variation of $\text{T-}\text{T}_{\text{r}}$.

**3.1.2 Thermodynamic Analysis and Experimental Methodology**

The ESA (αₑ) determination was achieved through a carefully controlled comparative methodology. By maintaining identical environmental conditions for both the fluorescent test specimen and non-fluorescent reference specimens, including: Solar irradiance ($\text{I}$), Convective heat transfer coefficient ($\text{h}_{\text{c}}$), Radiative heat transfer coefficient ($\text{h}_{\text{r}}$), Air temperature ($\text{T}_{\text{a}}$), Radiative exchange temperature ($\text{T}_{\text{r}}$).

We enabled accurate determination of the unknown ESA through temperature interpolation between the test specimen and reference specimens with known solar absorptivities (αₑ). This approach ensures measurement consistency while eliminating environmental variability as an error source. To determine the ESA ($\text{α}_{\text{e}}$) of fluorescent coolers, we employed a comparative methodology using:

a. A non-fluorescent reference sample (named as Sample 1). The same sample was irradiated with solar simulation through a 400 nm long-pass filter (UV blocked). Under this condition, no fluorescence is excited.

b. A fluorescent radiative cooler sample (named as Sample 2). The sample was irradiated with full-spectrum solar simulation (including UV, no filter). Under this condition, the PEO/Clay-UP component in the material absorbs UV photons and emits visible fluorescence.

Both were analyzed under identical conditions in our indoor solar simulator setup, where the equality $T_{r}$=$T_{a}$was strictly maintained.

So,

$$\begin{aligned} \text{α}_{\text{e}}\text{I=}\text{h}_{\text{r}}\left( \text{T-}\text{T}_{\text{a}} \right)\#\left( \text{S}\text{10} \right) \end{aligned}$$

The energy balance for the non-fluorescent sample (Sample 1) simplifies to $\text{Q}_{\text{absorbed}}\text{=}\text{Q}_{\text{thermal}}$ , with all absorbed energy converting to heat, yielding a temperature rise $\text{∆}\text{T}_{\text{1}}\text{= }\text{T}_{\text{1}}\text{-}\text{T}_{\text{a}}$. For the fluorescent sample (Sample 2), the balance becomes $\text{Q}_{\text{absorbed}}\text{=}\text{Q}_{\text{thermal}}\text{+}\text{Q}_{\text{fluorescent}}$, where fluorescence emission reduces the thermal component, resulting in a lower temperature rise ​$\text{∆}\text{T}_{\text{2}}\text{= }\text{T}_{\text{2}}\text{-}\text{T}_{\text{a}}$.

Assuming identical thermal conductivity and dissipation (governed by Newton’s cooling law,

$$\begin{aligned} \text{P}_{\text{thermal}}\text{=κ∆T}\#\left( \text{S}\text{11} \right) \end{aligned}$$

where κ is the heat dissipation coefficient. The temperature difference ratio of the two samples reflects the ratio of absorbed energy:

$$\begin{aligned} \frac{\text{ESA}_{\text{2}}}{\text{ESA}_{\text{1}}}\text{=}\frac{\text{Δ}\text{T}_{\text{2}}}{\text{Δ}\text{T}_{\text{1}}}\#\left( \text{S1}\text{2} \right) \end{aligned}$$

the ESA is proportional to the temperature differences:

$$\begin{aligned} \text{ESA}_{\text{2}}\text{=}\frac{\text{Δ}\text{T}_{\text{2}}}{\text{Δ}\text{T}_{\text{1}}}\text{×}\text{ESA}_{\text{1}}\#\left( \text{S1}\text{3} \right) \end{aligned}$$

Since Sample 1 exhibits no fluorescence, so, $\text{ESR}_{\text{1}}\text{=}\text{R}_{\text{1}}$; $\text{ESA}_{\text{1}}\text{=}\text{A}_{\text{1}}$

$$\begin{aligned} \text{ESR}_{\text{2}}\text{=1-}\text{ESA}_{\text{2}}\text{-T≈}\text{R}_{\text{1}}\text{+}\left( \text{1-}\frac{\text{T}_{\text{2}}\text{-}\text{T}_{\text{ambient}}}{\text{T}_{\text{1}}\text{-}\text{T}_{\text{ambient}}} \right)\text{A}_{\text{1}}\#\left( \text{S1}\text{4} \right) \end{aligned}$$

First, both samples were exposed to a solar irradiance of 1000 W/m^2^ for approximately 60 minutes to be heated under the solar simulator. The same samples were irradiated by a solar simulator with and without a 400 nm optical filter to investigate the temperature reduction effect induced by fluorescence emission. After this exposure, the solar simulator was turned off, and the samples were allowed to cool down naturally in the ambient air. The temperature changes of the samples during this period were recorded and are shown in **Figure S22**. All measurements were conducted in a custom-built radiative cooling chamber, which effectively eliminates heat convection and conduction, ensuring that the observed temperature differences arise primarily from radiative heat transfer processes.

Based on this analysis, the ESA of the fluorescent radiative cooler can be quantitatively determined through measured temperature differentials under controlled solar irradiance. Finally, the true fluorescence contribution (ESR-SR) also can be obtained. This methodology provides a robust estimation while effectively decoupling fluorescence effects from other thermal influences.


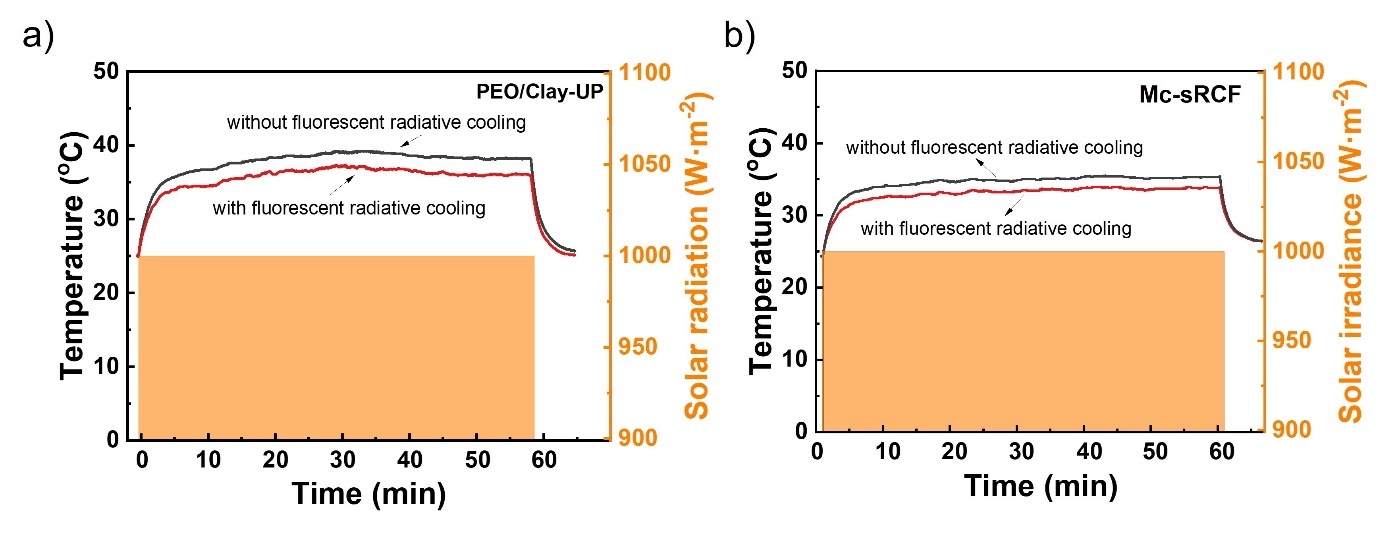


**Figure S22.** Temperature evolution of **a)** PEO/Clay-UP and **b)** Mc-sRCF samples with and without fluorescent radiative cooling under 1000 W/m² solar irradiance.

Notably, in our material system, when the PEO/Clay-UP component absorbs UV photons, the energy follows a preferential pathway:(1) Most of the absorbed UV energy is converted into visible fluorescence (around 440 nm) through radiative transition. These fluorescent photons escape the material entirely and do not contribute to heating. (2) Only a minimal fraction of the absorbed energy is converted into heat through non-radiative relaxation. This "fluorescence-dominated" energy dissipation mechanism means that the contribution of UV light to material heating is suppressed to a minimum. The majority of absorbed UV energy leaves the material as photons, not as heat.

From the perspective of surface energy balance, escaping fluorescent photons have the same effect as reflected sunlight, as both carry energy away without contributing to heating. Therefore, the effective solar reflectance of the material under the red-line condition is enhanced by the energy carried away by fluorescence. Since most absorbed ultraviolet energy is dissipated through fluorescence rather than converted to heat, the net additional heating contributed by ultraviolet light in the red-line condition is negligible. Meanwhile, the energy emitted through fluorescence effectively enhances the overall reflectance of the material, providing an additional pathway for heat dissipation. Compared to the black reference condition, this results in a lower net solar absorptance (thermal), thereby leading to a reduction in the equilibrium temperature.

Therefore, the data in **Figure S22** demonstrates that through a fluorescence-dominated energy dissipation mechanism, the material converts most absorbed ultraviolet energy into escaping visible photons, equivalently increasing its effective solar reflectance and achieving reduced net heat absorption and lower equilibrium temperature. **3.2. Calculation of the specific surface area for fabrics**

The standard form of the BET equation is:

$$\begin{aligned} \frac{\frac{\text{P}}{\text{P}_{\text{0}}}}{\text{V}\left( \text{1-}\frac{\text{P}}{\text{P}_{\text{0}}} \right)}\text{=}\frac{\text{1}}{\text{V}_{\text{m}}\text{×C}}\text{+}\frac{\text{C-1}}{\text{V}_{\text{m}}\text{×C}}\text{∙}\frac{\text{P}}{\text{P}_{\text{0}}}\#\left（ \text{S1}\text{5} \right） \end{aligned}$$

$$\begin{aligned} \text{V}_{\text{m}}\text{=}\frac{\text{1}}{\text{a+b}}\#\left（ \text{S1}\text{6} \right） \end{aligned}$$

$$\begin{aligned} \text{C=}\frac{\text{1}}{\text{V}_{\text{m}}\text{×b}}\#\left（ \text{S1}\text{7} \right） \end{aligned}$$

$$\begin{aligned} \text{S=}\text{N}_{\text{A}}\text{×}\frac{\text{a}_{\text{m}}}{\text{22414}}\text{×}\text{V}_{\text{m}}\#\left( \text{S1}\text{8} \right) \end{aligned}$$

Where:

- $\text{P}_{\text{0}}$– Saturation vapor pressure of the adsorbate at the adsorption temperature
- $\text{V}_{\text{m}}$– Monolayer saturation adsorption capacity of the adsorbent for the adsorbate (cm³/g)
- $\text{C}$– BET constant related to the adsorption energy of the adsorbent
- $\text{S}$– Specific surface area (m²/g)
- $\text{a}_{\text{m}}$– Cross-sectional area of an adsorbate molecule (m²)
- $\text{22414}$– Volume occupied by 1 mole of gas at standard temperature and pressure (STP, cm³)
- $\frac{\text{P}}{\text{P}_{\text{0}}}$– Relative pressure
- $\text{P}$– Equilibrium adsorption pressure (bar)
- $\text{V}$– Adsorption amount of the adsorbate on the adsorbent at equilibrium pressure P(cm³/g)
- $\text{a}$– Slope of the fitted straight line
- $\text{b}$– Intercept of the fitted straight line
- $\text{N}_{\text{A}}$– Avogadro’s constant (6.02×10^23^)

Based on BET equation (S15) - (S18) above, the BET Surface Area of three samples: PEO/Clay-UP, PVDF/TiO_2_, Mc-sRCF can be analyzed and plotted in **Figure S23**, the date can be listed in Supplementary **Table S1**.


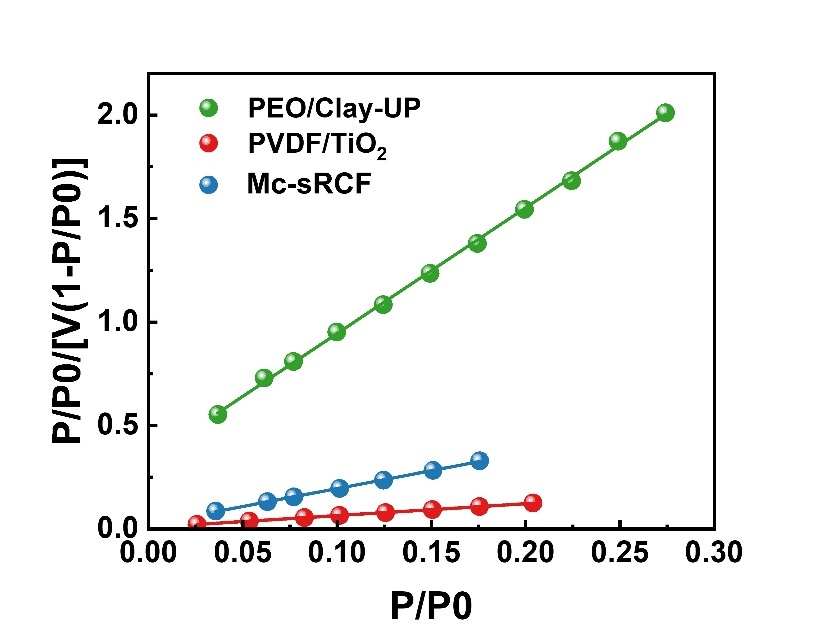


**Figure S23.** BET Multipoint Method to Fit Line

**4. Supplementary Tables**

**Table S1. BET Surface Area Analysis (Multipoint Method)**

| **Sample: PEO/Clay-UP** | | | |
| --- | --- | --- | --- |
| Slope a | 6.075058 | Intercept b | 0.3371290 |
| Correlation coefficient r | 0.99958 | BET constant C | 19.0 |
| P/P0 point range | 0.0368～0.2742 | Monolayer adsorption capacity Vm | 0.1560 cm³/g |
| BET Specific Surface Area | | | **0.6786 m^2^/g** |
| **Sample: PVDF/TiO_2_** | | | |
| Slope a | 0.575040 | Intercept b | 0.0072792 |
| Correlation coefficient r | 0.99988 | BET constant C | 80.0 |
| P/P0 point range | 0.0257～0.2039 | Monolayer adsorption capacity Vm | 1.7173 cm³/g |
| BET Specific Surface Area | | | **7.4719 m^2^/g** |
| **Sample: Mc-sRCF** | | | |
| Slope a | 1.728302 | Intercept b | 0.0226179 |
| Correlation coefficient r | 0.99972 | BET constant C | 77.4 |
| P/P0 point range | 0.0356～0.1757 | Monolayer adsorption capacity Vm | 0.5711 cm³/g |
| BET Specific Surface Area | | | **2.4850 m^2^/g** |

**Table S2. Review of Cooling Performance in Advanced Photoluminescent Radiative Coolers.**

| Sample | Spectral  performance | | Radiation Cooling performance | | Photoluminescence Contribution | | References |
| --- | --- | --- | --- | --- | --- | --- | --- |
|  | Solar reflectivity  (%) | Mid-infrared emissivity  (%) | Temperature drops  (^o^C) | Cooling power  (W·m^-2^) | Δ Temperature drops  (^o^C) | Δ Cooling power  (W·m^-2^) |  |
| PCRCs | over 90 | 95 | 2.2-5.4 | 25.6-51.7 |  |  | [2] |
| WC-RCE | 91.68-95.95 | 90.71 | 2.48 | 61.57 | 0.38 | 1.40 | [3] |
| Fluorescent materials | 89.8-92.6 | 90.1-90.9 | 0.8-1.5 |  |  |  | [4] |
| SDRC | 93.4 | 96 | 3.3 | 64.5 |  |  | [5] |
| GE-DNA aerogel | 104.0 (0.4-0.8 μm) | 90 | 2.9-16.0 |  |  |  | [6] |
| MHM | 93 | over 96 | 3.9 | 69.0 |  |  | [7] |
| SARC | 95 |  | 3.3-7.6 | 45.2-66.5 |  |  | [8] |
| PFTPCs | 93-95.8 | over 96 | 2.9-3.4 |  | 0-2.1 |  | [9] |
| CRC | 88.7-93.3 | 94 | 4-15 |  |  |  | [10] |
| HSCF | 89-93 | 98-99 | 4.7-5.8 |  |  |  | [11] |
| Mc-sRCF  This work | 93.45 | 95.34 | 3.9-10 | 83.78 | 1.1 | 2.88 |  |

**5. Supplementary Movies**

**Movie S1: Evaluation of Anti-Soiling Property in PEO/Clay-UP fabric.**

**Movie S2:** **Evaluation of Anti-Soiling Property in Mc-sRCF.**

**Movie S3:** **Evaluation of Flame Retardancy in PEO/Clay-UP fabric.**

**Movie S4:** **Evaluation of Flame Retardancy in Mc-sRCF.**

**6. References in** **Supporting Information**

[1] H. Liu, Y. Wang, W. Sun, M. Chen, Z. Chen, Y. Hou, Y. Zheng, B. Yu, *Small* **2025**, 21, 2505210.

[2] X. Wang, Q. Zhang, S. Wang, C. Jin, B. Zhu, Y. Su, X. Dong, J. Liang, Z. Lu, L. Zhou, W. Li, S. Zhu, J. Zhu, *Science Bulletin* **2022**, 67, 1874.

[3] S. Son, S. Jeon, J. H. Bae, S. Y. Lee, D. Chae, J. Y. Chae, T. Paik, H. Lee, S. J. Oh, *Materials Today Physics* **2021**, 21, 100496.

[4] X. Ma, Y. Fu, D. Liu, N. Yang, J.-G. Dai, D. Lei, *Advanced Optical Materials* **2024**, 12, 2303296.

[5] X. Xue, M. Qiu, Y. Li, Q. M. Zhang, S. Li, Z. Yang, C. Feng, W. Zhang, J.-G. Dai, D. Lei, W. Jin, L. Xu, T. Zhang, J. Qin, H. Wang, S. Fan, *Advanced Materials* **2020**, 32, 1906751.

[6] J.-W. Ma, F.-R. Zeng, X.-C. Lin, Y.-Q. Wang, Y.-H. Ma, X.-X. Jia, J.-C. Zhang, B.-W. Liu, Y.-Z. Wang, H.-B. Zhao, *Science* **2024**, 385, 68.

[7] J. Huang, D. Fan, *Journal of Alloys and Compounds* **2022**, 924, 166480.

[8] Q. Gong, H. Chun Wong, J. Chen, P. Li, L. Lu, *Chemical Engineering Journal* **2024**, 494, 153262.

[9] T. Wang, Y. Liu, Q. Xuan, X. Ma, Y. Fang, Y. Dong, D. Lei, J.-G. Dai, *Advanced Science* **2025**, 12, e11599.

[10] D. Feng, A. S. Witty, F. I. Birnbaum, O. G. R. Gonzalez, A. Felicelli, W.-J. Lee, E. C. Barber, X. Ruan, *Advanced Materia*ls **2025**, 37, e04382.

[11] J. He, Y. Chen, R. Guo, S. Tang, *ACS Nano* **2025**, 19, 30361.
